# Nutrient Intake and Status in Adults Consuming Plant-Based Diets Compared to Meat-Eaters: A Systematic Review

**DOI:** 10.3390/nu14010029

**Published:** 2021-12-23

**Authors:** Nicole Neufingerl, Ans Eilander

**Affiliations:** Unilever Foods Innovation Centre, 6708 WH Wageningen, The Netherlands; nicole.neufingerl@unilever.com

**Keywords:** plant-based diet, dietary intake, micronutrients, nutritional status, vegetarian, vegan

## Abstract

Health authorities increasingly recommend a more plant-based diet, rich in fruits, vegetables, pulses, whole grains and nuts, low in red meat and moderate in dairy, eggs, poultry and fish which will be beneficial for both health and the environment. A systematic review of observational and intervention studies published between 2000 and January 2020 was conducted to assess nutrient intake and status in adult populations consuming plant-based diets (mainly vegetarian and vegan) with that of meat-eaters. Mean intake of nutrients were calculated and benchmarked to dietary reference values. For micronutrient status, mean concentrations of biomarkers were calculated and compared across diet groups. A total of 141 studies were included, mostly from Europe, South/East Asia, and North America. Protein intake was lower in people following plant-based diets compared to meat-eaters, but well within recommended intake levels. While fiber, polyunsaturated fatty acids (PUFA), folate, vitamin C, E and magnesium intake was higher, eicosapentaenoic acid (EPA) and docosahexaenoic acid (DHA) intake was lower in vegetarians and vegans as compared to meat-eaters. Intake and status of vitamin B12, vitamin D, iron, zinc, iodine, calcium and bone turnover markers were generally lower in plant-based dietary patterns compared to meat-eaters. Vegans had the lowest vitamin B12, calcium and iodine intake, and also lower iodine status and lower bone mineral density. Meat-eaters were at risk of inadequate intakes of fiber, PUFA, α-linolenic acid (ALA), folate, vitamin D, E, calcium and magnesium. There were nutrient inadequacies across all dietary patterns, including vegan, vegetarian and meat-based diets. As plant-based diets are generally better for health and the environment, public health strategies should facilitate the transition to a balanced diet with more diverse nutrient-dense plant foods through consumer education, food fortification and possibly supplementation.

## 1. Introduction

Our current food system is not sustainable as global food production is threatening climate stability and ecosystem resilience. In addition, a large part of the world’s population is suffering from malnutrition, as one in every nine people is undernourished or hungry, one in three people is overweight or obese and 2 billion people are estimated to suffer from micronutrient deficiencies [1]. Unhealthy diets are a major cause of malnutrition and both are among the top ten risk factors contributing to the global burden of disease [2].

Globally, many governmental bodies and health authorities recognize the urgency to tackle this problem. The second goal of the United Nation’s Sustainable Development Goals aims to end hunger, achieve food security and improved nutrition and promote sustainable agriculture [3]. In 2019, The EAT Lancet report advocated on the importance of food as the single strongest lever to optimize human health and environmental sustainability on Earth and proposed a planetary health diet as sustainable solution [4]. Similarly, guiding principles for sustainable and healthy diets by the Food and Agriculture Organization of the United Nations (FAO) and World Health Organization (WHO) were launched in the same year [5]. Both reports recommend a balanced diet rich in fruits, vegetables, pulses, whole grains and nuts, with some fish, eggs, poultry and dairy, but limited in red meat and starchy vegetables. These plant-based diets can include different forms such as semi-vegetarian, flexitarian, pesco-vegetarian, lacto-ovo-vegetarian and vegan diets.

It is estimated that globally shifting from current diets to plant-based diets, will lower the risk of premature mortality from non-communicable diseases by 18–21% and reduce green-house-gas emissions by 54–87% [6]. Among dietary factors, high intake of sodium, low intake of whole grains, fruits, nuts and seeds and vegetables were among the top five dietary risk factors for deaths and disability adjusted life years (i.e., DALYs) associated with cardiovascular diseases, cancers and type 2 diabetes globally and in many countries [7].

While plant-based diets are considered healthier, they need to be balanced and diverse in order to provide the right amount of nutrients daily required for a healthy life. Previous reviews have indicated that vegetarians and vegans may risk vitamin B12, vitamin D, iron, zinc and calcium deficiency as these micronutrients can mostly be found in animal foods or have a lower bioavailability in plant foods [8,9,10,11,12,13,14]. Additionally, the intake of eicosapentaenoic acid (EPA) and docosahexaenoic acid (DHA) which are mainly present in fish and seafood has been shown to be inadequate in vegetarians and vegans [15,16]. However, an evaluation on dietary intake and nutritional status of a wider range of nutrients in populations consuming a plant-based diet is currently lacking.

Therefore, we aimed to conduct a systematic literature review to assess the intake and status of energy, macro- and micronutrient status of adults consuming a plant-based diet and to compare these with those of meat-eaters.

## 2. Materials and Methods

### 2.1. Search Strategy

We used a systematic approach to select studies comparing energy and nutrient intake and/or status of adults consuming plant-based diets, including studies that compared these data with adults consuming diets with meat. We systematically searched PubMed database using a search string that included different terms for plant-based diets, in combination with terms on dietary intake or nutritional status, along with predefined nutrients of specific interest, i.e., (diet OR intake OR “nutritional status” OR adequacy OR deficien*) in the title or abstract AND (vegetarian OR pescatarian OR vegan OR flexitarian OR meat?free OR “less meat” OR no?meat OR dairy?free OR no?dairy OR plant?based OR plant?forward OR sustainable) in title or abstract AND (nutrient* OR vitamin* OR mineral* OR micronutrient* OR zinc OR iodine OR iron OR calcium OR thiamin? OR riboflavin OR niacin OR “pantothenic acid” OR pyridoxin OR biotin OR “folic acid” OR folate OR cobalamin OR retinol OR caroten* OR “omega-3 fatty acid” OR “fish fatty acid*” OR PUFA OR “polyunsaturated fatty acid*” OR DHA OR “docosahexaenoic acid” OR “eicosapentaenoic acid” OR EPA OR an?emi*) in all fields).

Reference lists of (systematic) reviews and meta-analyses of interest were checked for additional studies. For the reporting of this systematic review the Preferred Reporting Items for Systematic reviews and Meta-Analyses (PRISMA) was used.

### 2.2. In- and Exclusion Criteria

- Type of studies: Observational studies and intervention studies (baseline data only), that compared nutrient intake and/or status of subjects following a predominantly plant-based diet with subjects following a conventional diet with meat were included. In addition, studies that reported only on subjects following a predominantly plant-based diet were also included. Generic reviews, case studies, and articles not published in English language were excluded;
- Diets: To be included in our review, studies had to report on voluntary self-selected diets with a primary focus on reducing animal food intake. Studies reporting on imposed or predesigned plant-based diets (e.g., marginal plant-based staple diets in developing countries, a prescribed vegetarian diet intervention, or modelled vegetarian diet scenario) were excluded, as well as articles on overly restrictive plant-based diets (e.g., raw food diet, macrobiotic diet), or healthy diets designed to lower non-communicable diseases (e.g., DASH diet, Mediterranean diet);
- Outcome parameters: Included studies provided data on either one or more of the following parameters: dietary intake of energy, protein, poly-unsaturated fatty acids (PUFA), α-linolenic acid (ALA), eicosapentanoic acid (EPA); docosahexanoic acid (DHA); dietary intake or nutritional status of micronutrients; bone markers;
- Study population: Generally healthy adult populations of 18 years and older. We excluded studies conducted in pregnant and lactating women, populations with specific diseases or in athletes;

The search was limited to literature published from 2000 until January 2020. Older articles were not included as they were not considered representative for current plant-based dietary patterns due to developments in the availability and range of plant-based products in recent decades.

### 2.3. Data Extraction

The identified articles were exported to an Endnote library and duplicates were removed. The titles and abstracts of the retrieved hits were screened for relevance by the two authors (AE, NN). Due to the vast amount of data obtained solely via references of reviews and meta-analyses, we decided to limit screening and full-text review of individual studies to articles published between January 2010 and January 2020.

For each study, we extracted information about population characteristics (age, gender), study location (country), reported diet patterns, in-/exclusion of supplement users and publication date. For each diet pattern, we extracted means, standard deviations (SD), standard errors (SE), medians, and 10th, 25th, 75th, 90th percentiles or ranges of parameters of dietary intake and nutritional status of the following nutrients: energy intake, protein, PUFA, total n-3 fatty acids, ALA, EPA, DHA, fiber, vitamin A, B1, B2, B6, B12, niacin, folate, vitamin C, D, E, iron, zinc, calcium, iodine, magnesium, and phosphorus. We also extracted data on prevalence of inadequate intake and prevalence of deficiencies of these nutrients and their corresponding cut-off criteria. In addition, data on hemoglobin, anemia and bone markers were collected for evaluation of iron and calcium status.

### 2.4. Data Handling

The definition and naming of vegetarian, vegan and other types of plant-based diets varied across studies. To ensure a consistent interpretation of the data, we applied the following uniform definitions to categorize all reported dietary patterns:

- Vegan: consuming meat, fish, dairy and eggs not at all/not during the days of dietary assessment OR ≤ once per month OR self-defined vegans;
- Vegetarian:consuming meat and fish not at all/not during the days of dietary assessment OR ≤ once per month OR self-defined vegetarians;
- Pesco-vegetarian: consuming meat not at all/not during the days of dietary assessment OR ≤ once per month OR self-defined;
- Semi-vegetarian: consuming meat (and fish) ≤ once per week but > once per month OR consuming meat (and fish) “seldom”/”occasionally”;
- Meat eating: consuming meat > once per week OR self-defined.

Some studies did not make a distinction between pesco-vegetarians and vegetarians, or between semi-vegetarians and vegetarians, or reported combined values for these groups. In these cases, the diets were categorized as “vegetarian”. Few studies reported on low/medium/high meat eaters. The cut-offs were differently defined per study, but these subjects generally consumed meat more than once per week. Two studies also reported on low/medium/high animal protein intakes. These categories were maintained.

If data were given as medians, interquartile ranges (IQR), SE or ranges, the data were converted into means and SD using standard formulas [17,18]. Because biomarkers and cut-off levels to define nutritional status and deficiencies varied across studies, only data that were based on definitions of the World Health Organization (WHO) or the Institute of Medicine (IOM) were included in our data analysis.

For articles that reported on intervention studies in subjects following a plant-based diet, only baseline data of dietary intake/status were used. If a study reported separate data for different subgroups following the same dietary pattern (e.g., based on sex, ethnicity or intervention treatment), the data were combined by taking weighted averages.

### 2.5. Data Analysis

Data were checked for correctness and outliers. Individual studies that reported extreme values (>1.5 × IQR) on intake or status for a specific nutrient were excluded from the analysis of this specific nutrient if the data seemed to be unreliable, i.e., if the mean value was derived based on calculations with extreme minimal or maximal values or if data were reported with presumably wrong units.

Separate analyses were carried out for studies that assessed nutrient intake from foods only and for studies that assessed nutrient intake from foods and supplements. For studies that reported on biomarkers of nutritional status, separate analyses were carried out for studies that excluded supplement users and for studies that did not exclude supplement users, i.e., the latter type of studies could include supplement users as well as non-users.

Average nutrient intake and status for the different dietary patterns were calculated across all studies and separately for studies including and excluding supplement use. Average micronutrient intakes of dietary patterns were compared with the estimated average requirements (EAR) [19,20]. Because of the lower bioavailability of iron and zinc from plant-based diets, iron and zinc intakes of vegans and vegetarians were compared to a bioavailability adjusted EAR, reflecting increased requirements according to the recommendation of the IOM [19]. In addition, per individual study, nutrient intake data of different dietary patterns were compared (i.e., meat-eating versus vegetarian; meat-eating versus vegan; vegetarian versus vegan). To account for the lower bioavailability of iron and zinc from plant-based diets when comparing intake among dietary patterns, intake data of vegetarians and vegans were adjusted by dividing reported iron intake by 1.8 and reported zinc intake by 1.5. 

Additionally, nutrient status data of the different dietary patterns were compared within studies using an independent sample *T*-tests. The percentage/number of studies with a significant difference between dietary patterns is reported. Additionally, the prevalence (range, mean) of nutrient deficiencies across studies was determined for the different dietary patterns.

## 3. Results

**Figure 1:**
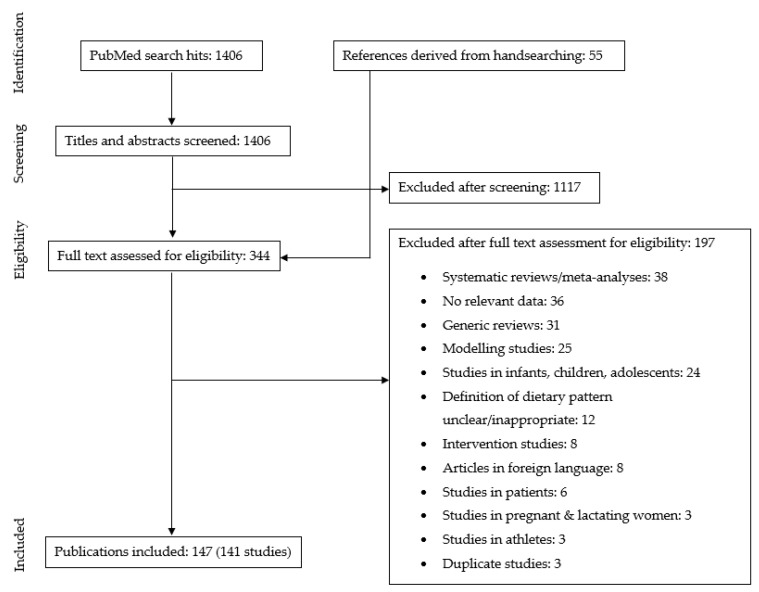
Flow diagram.

The initial literature search retrieved 1406 hits. After initial screening of titles and abstracts of all retrieved hits, 344 articles remained. These articles were read in full to assess their eligibility. Among these articles were 38 relevant reviews and meta-analyses, from which we derived an additional 55 references via handsearching of the reference list. After the full-text screening of the articles, a total of 147 articles reporting on 141 individual studies were included. See Figure 1 for more details of the screening process.

Most studies were conducted in Europe, South/East Asia and North America. There were hardly any studies from South America or West Asian countries and no studies from Africa. Twelve studies were conducted in postmenopausal women or older populations >60 years of age. Vegetarian and meat-eating dietary patterns were most reported on, less studies reported on vegan (*n* = 64), and only few on pesco-or semi-vegetarian diets. Most studies reported on intake of protein (n = 64), calcium (*n* = 40), vitamin B12 (*n* = 39) and iron (*n* = 38), only few on ALA (*n* = 9), EPA and DHA (*n* = 8), or iodine (*n* = 5). Biomarker data on nutritional status were mostly available for vitamin B12 (*n* = 48), folate (*n* = 40), iron and hemoglobin (*n* = 17). Seventeen studies provided unreliable intake or status data for one or more of the reported nutrients. Therefore, these data were excluded for the analysis of these specific nutrients. For an overview of study characteristics see Table 1; see Supplementary Table S1 for details of the included individual studies.

### 3.1. Energy, Protein, Fiber and Fatty Acids

#### 3.1.1. Energy

Sixty-five studies reported on energy intake. Average energy intake was similar across all dietary patterns with mean (minimum, maximum) intakes of 2101 (1374, 2985) kcal/d for meat-eaters 1947 (1130, 2757) for vegans and 2098 (1495, 2820) in vegetarians.

#### 3.1.2. Protein

**Figure 2:**
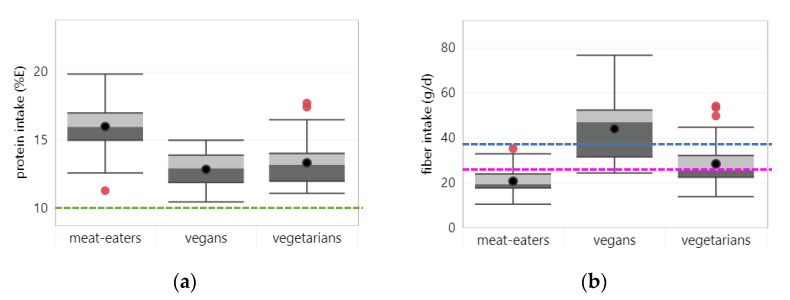

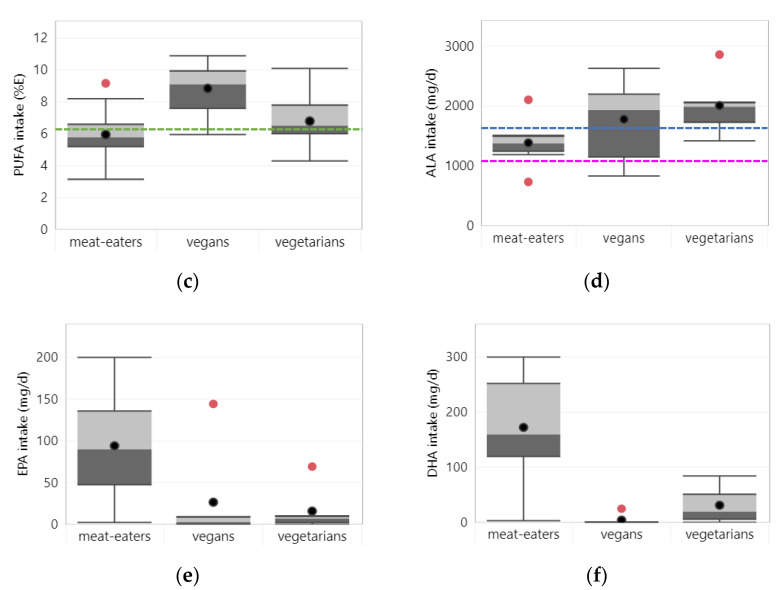
Boxplots represent 25, 50 and 75 percentiles of intake with whiskers at <1.5 interquartile range (IQR) per diet group; black dots represent mean intake and red dots outliers >1.5 IQR: (**a**) protein—dotted lines represent the lower limit of the acceptable macronutrient distribution range; (**b**) fiber—blue and pink dotted lines represent adequate intakes for men and women respectively; (**c**) polyunsaturated fatty acids (PUFA)—dotted line represents lower acceptable macronutrient distribution range; (**d**) α-linolenic acid (ALA)—blue and pink dotted lines represent adequate intakes for men and women respectively; (**e**) eicosapentaenoic acid (EPA); (**f**) docosahexaenoic acid (DHA).

Sixty-four studies reported on protein intake, of which 50 studies assessed intake from foods only. Across all studies, average protein intake was lower in vegetarians (13.4% E) and vegans (12.9% E) compared to meat eaters (16.0% E), irrespective of whether intake from supplements was assessed (see Figure 2a). Average protein intakes were above the lower limit of the acceptable macronutrient distribution range (AMDR) (i.e., 10%E). None of the studies reported protein intakes below the AMDR for any dietary pattern. For more information see Supplementary Figure S1 on mean nutrient intakes across dietary patterns for studies that assessed nutrient intakes from foods only and for studies assessing nutrient intake from foods and supplements.

#### 3.1.3. Fiber

Forty-three studies reported on fiber intake, of which 35 studies considered fiber intake from foods only. Across all studies, average fiber intake was highest in vegans (44 g/d), followed by vegetarians (28 g/d) and lowest in meat-eaters (21 g/d) (see Figure 2b). The same order was observed irrespective of whether intake from supplements was assessed. The average fiber intake of vegans met the adequate intake (AI), while for meat-eaters it was below the AI. The average fiber intake of vegetarians was sufficient to meet the AI for women, but not for men. Looking at individual studies, 74% (14/19 studies) reported fiber intakes of vegans met the AI compared to 29% (10/35 studies) in vegetarians and 6% (2/33 studies) in meat-eaters.

#### 3.1.4. PUFA

Thirty-six studies reported on PUFA intake, of which 31 assessed intake from foods only. Across all studies, average PUFA intake was highest in vegans (8.84% E), and lowest in meat-eaters (5.95% E), with pesco-vegetarians (7.77% E), semi-vegetarians (7.67% E) and vegetarians (6.79% E) in between (see Figure 2c). Similar patterns were observed in studies that did and did not assess intake from supplements. Average PUFA intake of meat-eaters was just below the lower AMDR (i.e., 6% E), while for all other dietary patterns mean intakes were above the lower AMDR.

#### 3.1.5. N-3 Fatty Acids

Twelve studies reported on intake of total n-3 fatty acids, nine reported on ALA intake, eight on EPA and DHA intake. All but three studies assessed intake from foods only. Across all studies, mean intake of total n-3 fatty acids tended to be higher in vegans (2.69 g/d) compared to vegetarians (1.36 g/d) and meat-eaters (1.08 g/d). Two studies also reported relatively high n-3 fatty acid intakes of 2.53 g/d in pesco- and 1.98 g/d in semi-vegetarians. The higher intake of n-3 fatty acids in plant-based dietary patterns was mainly due to higher intakes of ALA in vegans (2.01 g/d) compared to vegetarians (1.78 g/d) and meat-eaters (1.38g/d) (see Figure 2d). On the other hand, intakes of EPA and DHA were considerably lower in vegans (27 and 4 mg/d) and vegetarians (16 and 31 mg/d) compared to meat-eaters (94 and 172 mg/d) (see Figure 2e,f). Pesco-vegetarians, as reported in one study of Adventists in the USA/Canada had highest DHA intakes (287 mg/d) [21].

While mean intake of ALA was above the AI for vegetarians and vegans, average combined intakes of EPA and DHA were below the lower AMDR (i.e., 250 mg/d). For meat-eaters, average EPA + DHA intake was in line with the AMDR, but average ALA intake failed to meet the AI for men.

Twenty-two studies reported on fatty acid status. Because of differences in methodologies used to measure fatty acid status, it was not possible to calculate overall means of fatty acid status but we describe the findings of individual studies that compared fatty acid status of dietary patterns. Six studies compared total PUFA status between diet groups. Most studies showed significantly higher PUFA status in vegetarians (3/3 studies) and vegans (3/5 studies) compared to meat-eaters. For ALA status, as reported in 11 studies, there was a significantly higher status in vegans (4/9 studies) and vegetarians (3/8 studies) compared to meat-eaters. Thirteen studies reported on EPA and/or DHA status, most of which reported lower EPA and DHA status in vegetarians (5/7 and 7/7 studies) and vegans (7/8 and 8/9 studies) compared to meat-eaters. Vegans also mostly had lower EPA and DHA status than vegetarians (5/6 and 5/6 studies).

### 3.2. Micronutrients

#### 3.2.1. Vitamin A

**Figure 3:**
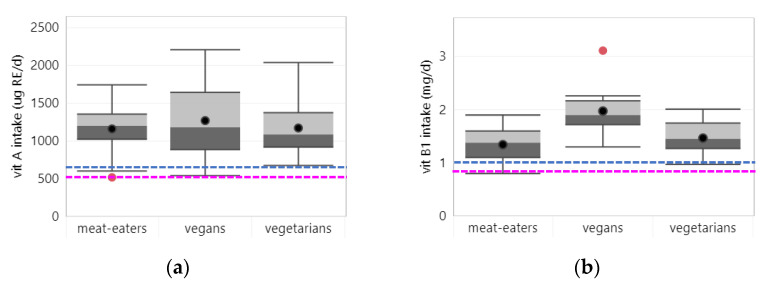

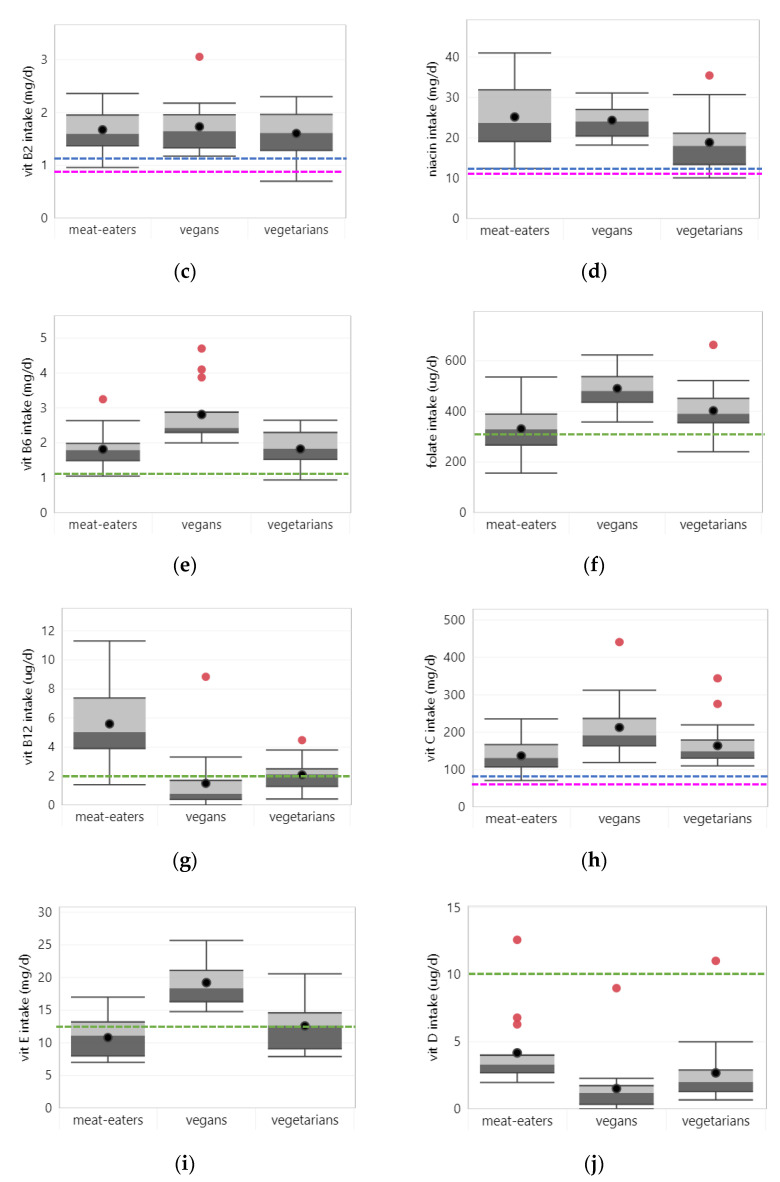
Vitamin intake per diet group in all studies. Boxplots show 25, 50 and 75 percentiles of intakes with whiskers at <1.5 interquartile range (IQR); black dots represent mean intake and red dots outliers >1.5 IQR; dotted lines represent the estimated average requirement (EAR) for adults (green), women (pink) and men (blue): (**a**) vitamin A; (**b**) vitamin B1; (**c**) vitamin B2; (**d**) niacin; (**e**) vitamin B6; (**f**) folate; (**g**) vitamin B12; (**h**) vitamin C; (**i**) vitamin D; (**j**) vitamin E.

Intake: Twenty-two studies reported on vitamin A intake, of which 17 considered intake from foods only. Across all studies, average vitamin A intake was similar across all dietary patterns (see Figure 3a). In two studies that assessed intake from foods and supplements, vegetarians tended to have a higher vitamin A intake [22,23]. For all dietary patterns, vitamin A intakes were well above the EAR (i.e., 500/625 µg RE for women/men). Only two studies (both considering intake from foods only) reported vitamin A intake below the EAR in meat-eaters in the US [24] and vegans in the UK [25].

Status: Five studies assessed beta-carotene status, of which three excluded supplement users. Across all studies, average beta-carotene status tended to be lower in vegetarians (0.4 µmol/L) compared to meat-eaters (0.8 µmol/L) and vegans (0.8 µmol/L). However, these differences were mainly influenced by one study among Finnish meat-eaters and vegans that reported relatively high beta-carotene levels for both dietary pattens [26]. In contrast, two studies that directly compared beta-carotene status between vegetarians and meat-eaters found significantly higher levels in vegetarians though [27,28]. See Supplementary Table S2 for descriptive data on nutritional status across dietary patterns for all studies, and separately for studies that did and did not exclude supplement users.

In addition, two studies, both excluding supplement users, assessed vitamin A status based on serum/plasma retinol levels, showing similar retinol levels across all dietary patterns (0.2.5/2.2/2.1 µmol/L in respectively meat-eaters, vegetarians, and vegans) [29,30]. Studies that compared status data between dietary patterns showed mixed results. Two out of three studies showed significantly lower retinol levels in vegetarians and vegans compared to omnivores [29,30]; while one study from India showed significantly higher retinol levels for vegetarians compared to omnivores [31]. For all dietary patterns, status data were well above the cut-off for vitamin A deficiency (i.e., retinol < 0.7 µmol/L).

#### 3.2.2. Vitamin B1

Intake: Twenty-three studies reported on vitamin B1 intake, of which 18 assessed intake from foods only. Across all studies, vegans tended to have a higher average vitamin B1 intake (1.97 mg/d) than vegetarians (1.47 mg/d) and meat-eaters (1.34 mg/d) (see Figure 3b). This was even more pronounced in studies that assessed intake from foods and supplements. Average vitamin B1 intake was above the EAR (i.e., 0.9/1.0 mg/d for women/men) for all dietary patterns. Yet, three studies from Taiwan [32], Japan [33] and Turkey [34] reported vitamin B1 intake below the EAR in meat-eaters only.

Status: A study from Switzerland assessed vitamin B1 status based on plasma levels, reporting somewhat higher levels in vegans (36.4 nmol/L) than in vegetarians (29.4 nmol/L) and meat-eaters (30.7 nmol/L) [30]. Another study from Austria reported a 2.5% prevalence of vitamin B1 deficiency (>25% Thiamine pyrophosphate effect) in meat-eaters with zero prevalence among vegetarians and vegans [35].

#### 3.2.3. Vitamin B2

Intake: Twenty-five studies reported on vitamin B2 intake, of which 19 assessed intake from foods only. Across all studies, average vitamin B2 intake was similar for all dietary patterns (see Figure 3c). In studies that assessed intake from foods and supplements, vegans and meat-eaters had slightly higher vitamin B2 intakes than vegetarians. For all dietary patterns, mean intake across all studies was above the EAR (i.e., 0.9/1.1 mg/d for women/men). Yet, three individual studies from Taiwan and Japan reported intakes below the EAR in vegetarians [32,36] and meat-eaters [33].

Status: One study from Switzerland assessed vitamin B2 status based on plasma levels, reporting somewhat higher levels in meat-eaters (92.0 nmol/L) than in vegetarians (82.4 nmol/L) and vegans (79.8 nmol/L) [30]. Another study from Austria reported on vitamin B2 deficiency (erythrocyte glutathione reductase activity coefficient >1.4), with prevalence of 33%, 12.5% and 10% in vegans, meat-eaters and vegetarians [35].

#### 3.2.4. Niacin

Intake: Twenty studies reported on niacin intake, of which 15 assessed intake from foods only. Across all studies, on average, vegetarians tended to have slightly lower niacin intake (18.8 mg/d) than vegans (24.3 mg/d) and meat-eaters (25.2 mg/d) (see Figure 3d). Mean intakes of niacin were higher in studies that assessed intake from foods and supplements, especially for meat-eaters and vegetarians. For all dietary patterns, mean intake across studies was above the EAR (i.e., 11/12 mg/d for women/men). Only two studies in Taiwanese adults [32] and Turkish women [34] reported niacin intakes of vegetarians below the EAR.

Status: One study from Switzerland assessed niacin status based on plasma levels, reporting lower levels in vegans (464 nmol/L) than in vegetarians (580 nmol/L) and meat-eaters (579 nmol/L) [30]. No studies reported on niacin deficiency based on the appropriate definition.

#### 3.2.5. Vitamin B6

Intake: Twenty-seven studies reported on vitamin B6 intake, of which 22 assessed intake from foods only. Across all studies, average vitamin B6 intake tended to be higher in vegans (2.81 mg/d) compared to vegetarians and meat-eaters (1.82 mg/d) (see Figure 3e), irrespective of whether studies assessed intake from supplements. Mean intakes were well above the EAR (i.e., 1.1 mg/d) for all dietary patterns. Only three studies from Taiwan and Japan showed mean intakes below the EAR in meat-eaters [33,37] or vegetarians [32,37].

Status: Eleven studies reported on vitamin B6 status using serum/plasma levels. Of these, five studies excluded supplement users. Across all studies, average vitamin B6 levels were similar for all dietary patterns. Additionally, most individual studies that compared vitamin B6 status between dietary patterns showed similar levels in vegetarians, vegans, and meat-eaters. Yet, three studies in Slovakia and Germany/the Netherlands showed significantly higher vitamin B6 levels in vegetarians compared to meat-eaters [28,38,39], while two studies in Taiwan showed significantly lower vitamin B6 levels in vegetarians compared to meat-eaters [32,37]. One Taiwanese study also assessed vitamin B6 deficiency (Plasma PLP < 20 nmol/L), reporting no deficiencies in vegetarians or meat-eaters [37].

#### 3.2.6. Folate

Intake: Thirty-four studies reported on folate intake, of which 27 assessed intake from foods only. Across all studies, vegans tended to have higher average folate intake (490 µg/d) than vegetarians (403 µg/d) and meat-eaters (331 µg/d) (see Figure 3f), irrespective of whether intake from supplements was assessed. Mean intakes were just above the EAR (i.e., 320 µg/d) in meat-eaters. While for vegetarians and vegans, 93–100% of individual studies (27/29 and 15/15 studies) reported folate intakes above the EAR, for meat-eaters 9 out of 24 studies (38%) found intakes below the EAR.

Status: Forty studies reported on folate status, of which 20 excluded supplement users. Across studies, folate status tended to be higher in plant-based dietary patters as compared to meat-eaters (19 nmol/L), with highest levels in vegans (29 nmol/L) and intermediate levels in vegetarians and semi-vegetarians (24 and 25 nmol/L). This order was similar in studies that included and excluded supplement users. Half of all studies (11/22 studies) that directly compared folate status between vegetarians and meat-eaters and three quarter of studies (9/12 studies) comparing vegans with meat-eaters, showed that meat-eaters had a significantly lower folate status. Eight studies assessed folate deficiency (<10 nmol/L in plasma/serum or <340 nmol/L in red blood cells) with average prevalence of 11% in meat-eaters, 0% in vegetarians and 1.5% in vegans.

#### 3.2.7. Vitamin B12

Intake: Thirty-nine studies reported on vitamin B12 intake, of which 32 assessed intake from foods only. Across all studies, average vitamin B12 intake was higher in meat-eaters (5.6 µg/d) compared to vegetarians (2.1 µg/d) and vegans (1.5 µg/d) (see Figure 3g). Studies that assessed intake from foods and supplements showed somewhat higher vitamin B12 intakes for all dietary patterns than studies that assessed intake from foods only. Yet intake of vegetarians and vegans remained clearly below that of meat-eaters. In studies that assessed intake from foods and supplements, all dietary patterns had a mean vitamin B12 intake above the EAR, though the median vitamin B12 intake of vegans was below the EAR (i.e., 2.0 µg/d). In studies that assessed intake from foods only, mean and median vitamin B12 intake of vegans was well below the EAR. Most individual studies that assessed intake from foods only (10/13 studies) reported a vitamin B12 intake below the EAR for vegans, and half of the studies did so for vegetarians. This indicates that vegans and vegetarians are at high risk of inadequate vitamin B12 intake when supplements are not considered.

Status: Vitamin B12 status was assessed in 48 studies based on serum or plasma vitamin B12 levels. Out of these, 26 studies excluded supplement users. Across all studies, mean vitamin B12 status tended to be higher in meat-eaters (309 pmol/L) than in vegetarians (220 pmol/L) and vegans (226 pmol/L). This was the case both in studies that included and excluded supplement users. Most studies that compared vitamin B12 status between dietary patterns, found significant lower status in vegetarians (22/31 studies) and vegans (8/15 studies) compared to meat-eaters. In studies that excluded supplement users, this became even more apparent. Vitamin B12 status in vegans and vegetarians was mostly similar (9/17 studies) or lower in vegans (7/17 studies). Thirteen studies assessed vitamin B12 deficiency (<150 pmol/L), of which 8 studies excluded supplement users. In meat-eaters, reported prevalence of vitamin B12 deficiency ranged between 0–16%, while in vegetarians, prevalence ranged from 0% in a national survey of the USA [40] up to 75% in a sample of Chinese older women [41], and in vegans from 4% in a local sample of Spanish adults [42] to 73% in a UK multi-center study [43]. Among studies that excluded supplement users, prevalence of vitamin B12 deficiency ranged between 4% and 70% for vegetarians in Germany and China, respectively; and between 6% and 7% for vegans, based on two studies from Spain and Germany [23,42,44].

#### 3.2.8. Vitamin C

Intake: Thirty-one studies reported on vitamin C intake, of which 26 assessed intake from foods only. Across all studies, average vitamin C intake was highest in vegans (213 mg/d), followed by vegetarians (166 mg/d) and then meat-eaters (137 mg/d) (see Figure 3h). The same order was observed irrespective of whether studies assessed intake from supplements. Average vitamin C intake of all dietary patterns was above the EAR. No single study reported intakes below the EAR (i.e., 60/75 mg for women/men).

Status: Seven studies reported on vitamin C status, using plasma levels, of which four studies excluded supplement users. Across all studies, average vitamin C levels were higher in vegetarians (62.7 µmol/L) and vegans (61.9 µmol/L) compared to meat-eaters (44.9 µmol/L). This was similar for studies including and excluding supplement users. All or most individual studies showed a significant higher vitamin C status in vegans (3/3 studies) and vegetarians (5/7 studies) compared to meat-eaters. Vegans as compared to vegetarians had similar (2/3 studies) or higher vitamin C status. Currently, there are no internationally accepted cut-off values to define vitamin C deficiency. One Swiss study reported on vitamin C deficiency defined as plasma vitamin C <11.1 µmol/L in men and <35.3 µmol/L in women, with 12% prevalence in meat-eaters and 4% prevalence in vegans and vegetarians [30].

#### 3.2.9. Vitamin D

Intake: Twenty-one studies reported on vitamin D intake, of which 15 assessed intake from foods only. Across all studies, average vitamin D intake tended to be highest in pesco-vegetarians (5.25 µg/d), followed by meat-eaters (4.17 µg/d), then vegetarians (2.67 µg/d), with lowest intakes in vegans (1.52 µg/d) (see Figure 3i). One study reported vitamin D intake ranging from 3.4 µg/d in meat eaters with a low meat intake to 4.0 µg/d in meat-eaters with a high meat-intake [45]. In studies that assessed intake from foods and supplements, vitamin D intake was generally higher for all dietary patterns than in studies that assessed intake from foods only. But the descending order of intake from pesco-vegetarians to vegans was maintained. Average vitamin D intake was below the EAR (i.e., 10 µg/d) in all dietary patterns indicating high prevalence of inadequate intakes in the overall population. Only one study among Adventist from the US/Canada, which assessed intake from foods and supplements, reported intakes above the EAR for pesco-vegetarians, meat-eaters, and vegetarians, but not vegans [21].

Status: Eleven studies reported on vitamin D status, based on serum/plasma 25(OH)D levels; five studies excluded supplement users. Across all studies, average vitamin D levels tended to be slightly higher in pesco-vegetarians (28.9 µg/L), meat-eaters (26.2 µg/L) and semi-vegetarians (25.8 µg/L) than in vegetarians (22.8 µg/L) and vegans (21.9 µg/L). Yet, data of pesco- and semi-vegetarians were only based on one study. Among studies that compared vitamin D status between dietary patterns, three out of nine showed significantly lower vitamin D status in vegetarians or vegans compared to meat-eaters. Vegans had similar vitamin D status as vegetarians in 5 out of 6 studies.

Four studies each reported on vitamin D insufficiency (25(OH)D < 20 µg/L) and vitamin D deficiency (25(OH)D < 10 µg/L). Reported prevalence of vitamin D deficiency in meat-eaters and pesco-vegetarians was low, ranging between 0 and 6% across studies. For vegetarians and vegans, vitamin D deficiency was much more prevalent, ranging between 0 and 33% in vegetarians and 3% and 67% in vegans. The highest prevalence of vitamin D deficiency in vegetarians and vegans was reported in a study among a sample of Finnish women [46]. Average prevalence of vitamin D insufficiency was 15% in meat-eaters and 25% in vegetarians and vegans. One study of Adventists in the USA/Canada also reported a high prevalence (41%) of vitamin D insufficiency in semi-vegetarians [47].

#### 3.2.10. Vitamin E

Intake: Eighteen studies reported on vitamin E intake, of which 14 assessed intake from foods only. Across all studies, average vitamin E intake tended to be higher in vegans (19.2 mg/d) compared to vegetarians (12.6 mg/d) and meat-eaters (10.8 mg/d) (see Figure 3j), irrespective of whether intake from supplements was assessed. Only for vegans, average vitamin E intake was well above the EAR (i.e., 12 mg/d), and all individual studies reported intakes above the EAR. Mean intake of vegetarians was just around the EAR, with six out of 14 studies reporting intakes below the EAR. Meat-eaters had an average vitamin E intake below the EAR with nine out of 14 studies reporting intakes that did not meet the EAR.

Status: Eight studies reported on vitamin E status, based on serum/plasma α-tocopherol levels; four studies excluded supplement users. Vitamin E status was similar across dietary patterns (25.4/25.5/20.5 µmol/L in meat-eaters, vegetarians, and vegans). Individual studies that compared vitamin E status between dietary patterns showed mixed results. Two out of six studies showed significant lower vitamin E levels in Swiss and Finnish vegans and/or vegetarians compared to meat-eaters [26,30], while two other studies showed significantly higher vitamin E levels in Slovakian vegans and/or vegetarians [27,48]. The latter studies included supplement users. One study assessed vitamin E deficiency (defined as plasma α-tocopherol < 13 µmol/L), reporting zero prevalence among meat-eaters and vegetarians and 3.8% among vegans [30].

### 3.3. Minerals

#### 3.3.1. Calcium

**Figure 4:**
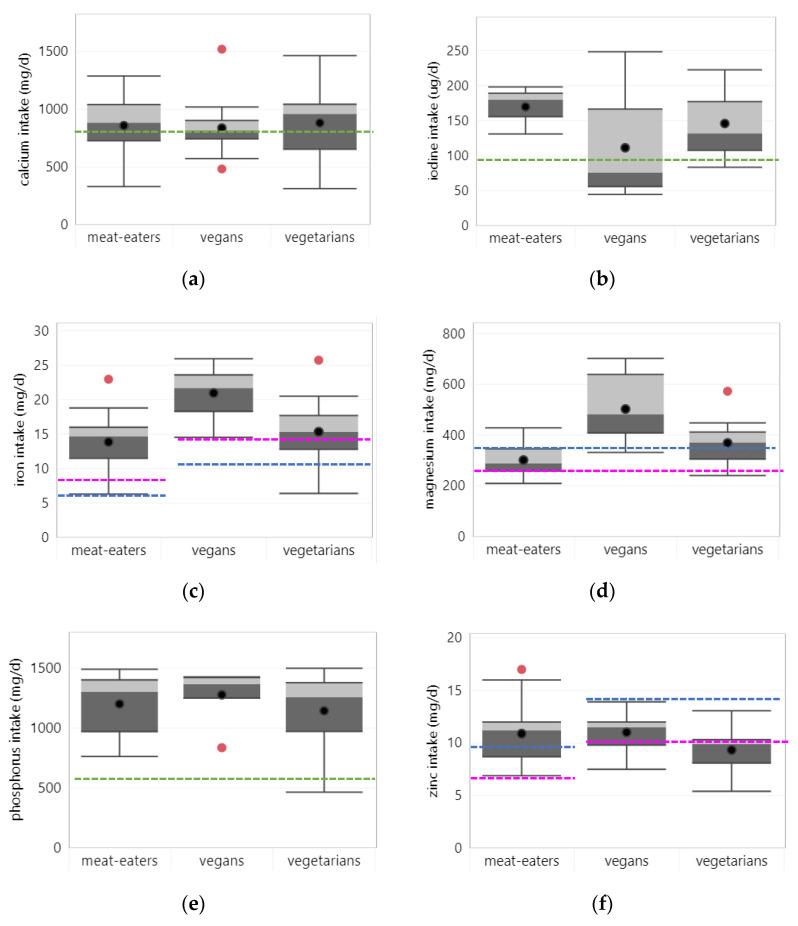
Mineral intake per diet group. Boxplots show 25, 50 and 75 percentiles of intakes with whiskers at <1.5 interquartile range (IQR); black dots represent mean intake and red dots outliers >1.5 IQR; dotted lines represent the estimated average requirement (EAR) for adults (green), women (pink) and men (blue): (**a**) calcium; (**b**) iron, EAR for vegans and vegetarians is adjusted for lower iron bioavailability; (**c**) iodine; (**d**) magnesium; (**e**) phosphorus; (**f**) zinc, EAR for vegans and vegetarians is adjusted for lower zinc bioavailability.

Intake: Forty studies reported on calcium intake, of which 33 assessed intake from foods only. Across all studies, average calcium intake was slightly higher in vegetarians (895 mg/d) than in vegans (838 mg/d) or meat-eaters (858 mg/d) (see Figure 4a), irrespective of whether intake from supplements was assessed. Mean intakes were (slightly) above the EAR (i.e., 800 mg/d) for all dietary patterns. One third of studies reported calcium intakes below the EAR in vegetarians (11/33 studies) and meat eaters (11/32 studies); vegans had a calcium intake below the EAR in seven out of 17 studies.

Status: Hormonal and bone turnover markers and bone mineral density were evaluated. Five studies reported on C-terminal telopeptide of type I collagen (CTX), four studies each reported on parathyroid hormone (PTH), osteocalcin (OC) and bone mineral density (BMD) of the lumbar spine, three on procollagen type 1 N-terminal propeptide (P1nP) and two on bone alkaline phosphatase (BAP). All but one study excluded supplement users. Across studies, average PTH levels tended to be higher in vegetarians (4.91 pmol/L) and vegans (5.21 pmol/L) than in meat-eaters (4.04 pmol/L). Also bone turnover markers tended to be higher in vegetarians and vegans as compared to meat-eaters, indicating accelerated bone turnover. Yet BMD of the lumbar spine was comparable between dietary patterns.

Five studies directly compared hormonal and bone turnover markers between vegetarians and meat-eaters; one study showed significantly higher PTH levels [49]; one significantly higher CTX and OC levels [50], and one significantly higher P1nP levels in vegetarians [50]. All three studies comparing vegans and meat-eaters showed significantly higher PTH [46,51], BAP [50,51] or CTX and OC levels [50] in vegans. Two out of three studies also showed significantly lower BMD of the lumbar spine in vegans compared to meat-eaters, while no significant differences were found between vegetarians and meat-eaters.

#### 3.3.2. Iodine

Intake: Five studies reported on iodine intake, of which three assessed intake from foods only. Across studies, average iodine intake tended to be lower in vegans (111 µg/d) and vegetarians (146 µg/d) than in meat-eaters (170 µg/d) (see Figure 4b). Mean iodine intakes were above the EAR (i.e., 95 µg/d) for all dietary patterns. However, three out of four studies (irrespective of whether intake from supplements was assessed), reported iodine intake of vegans below the EAR [52,53,54], suggesting inadequate iodine intakes may be highly prevalent in vegan populations.

Status: Five studies reported on iodine status, none of which excluded supplement users. Across studies, average iodine status was similar between meat-eaters (111 µg/L), vegetarians (103 µg/L) and vegans (105 µg/L). Yet, most studies that directly compared iodine status across diet groups showed a significantly lower iodine status in vegetarians (2/3 studies) and vegans (3/3 studies) compared to meat eaters. Three studies reported on iodine deficiency (urinary iodine < 100 µg/L) with on average very high prevalence in vegans (92%) and somewhat lower prevalence in meat-eaters (51%) and vegetarians (37%).

#### 3.3.3. Iron

Intake: Thirty-eight studies reported on iron intake, of which 30 assessed intake from foods only. Across studies, average iron intake tended to be higher in vegans (21.0 mg/d) compared to vegetarians (15.3 mg/d) and meat eaters (13.9 mg/d) (see Figure 4c), independent of whether intake from supplements was assessed. Mean iron intakes were above the (bioavailability-adjusted) EAR in all diet groups. However, vegetarians failed to meet the bioavailability-adjusted EAR (i.e., 14.6/10.8 mg/d in women/men) in seven out of 32 studies. 

Status: Seventeen studies reported iron status based on serum or plasma ferritin values, of which ten excluded supplement users. Iron status tended to be higher in meat-eaters (55.5 µg/L) than in vegetarians (33.8 µg/L) and vegans (31.3 µg/L) and was particularly low in vegetarian women (24.3 µg/L). About half of all studies (6/13 studies) that directly compared iron status between meat eaters and vegetarians and two out of three studies comparing meat-eaters and vegans, showed that meat-eaters had a significantly higher iron status. In studies that excluded supplement users, this was the case even more so. A National dietary Survey from China showed that iron status was also significantly higher among the highest quintile of animal protein consumers compared to the lowest quintile [55]. Four studies assessed iron deficiency (ferritin < 15 µg/L) with average prevalence of 7% in meat-eaters, 11% in vegetarians and 15% in vegans. Mean hemoglobin values were similar and adequate across diet patterns (i.e., 139/136/140 g/L for meat-eater, vegans and vegetarians, respectively). Only one out of 17 studies showed significantly lower hemoglobin status in Australian vegetarians and vegans compared to meat-eaters [29]. Yet, studies that assessed anemia (hemoglobin <120/130 g/L in women/men), reported higher average prevalence numbers in vegetarians and vegans (11 and 17%, respectively) than in meat eaters (5%).

#### 3.3.4. Magnesium

Intake: Twenty-six studies reported on magnesium intake, of which 21 assessed intake from foods only. Across all studies, average magnesium intake was higher in vegans (503 mg/d) than in vegetarians (373 mg/d) and meat-eaters (302 mg/d) (see Figure 4d). In studies that assessed intake from foods only, magnesium intake was somewhat lower across all groups than in studies that assessed intake from foods and supplements, but vegans had still the highest intake. Average magnesium intake of vegans and vegetarians was above the EAR (307.5 md/g), while for meat-eaters, intake did not meet the EAR for men. More than half of individual studies (11/19 studies) reported magnesium intake of meat-eaters to be below the EAR, while for vegetarians and vegans most studies (16/22 and 10/10 studies, respectively) reported intakes above the EAR.

Status: Four studies reported on magnesium status, three of which excluded supplement users and did not show significant differences among diet groups.

#### 3.3.5. Phosphorus

Intake: Eighteen studies reported on phosphorus intake, of which 14 assessed intake from foods only. Across all studies, average phosphorus intake was similar between dietary patterns (see Figure 4e). Average phosphorus intake was somewhat higher in studies that assessed intake from foods and supplements, especially among vegetarians. Average phosphorus intake was well above the EAR (i.e., 580 mg/d) for all dietary patterns. Only one study reported phosphorus intake below the EAR in a sample of vegetarian Buddhist nuns in Vietnam [56].

Status: Two studies (both excluding supplement users) reported on phosphorus status with similar levels in vegetarians (111 mmol/L) and meat-eaters (120 mmol/L).

#### 3.3.6. Zinc

Intake: Thirty-one studies reported on zinc intake, of which 23 assessed intake from foods only. Irrespective of supplement use, mean zinc intake across studies was similar across dietary patterns (see Figure 4f). Yet, when comparing intakes with the EAR, adjusted for the lower bioavailability of zinc from plant-based diets (i.e., 10.3/14.3 mg/d for women/men), vegetarians and vegans had mean zinc intakes below the EAR (for men). This suggests inadequate zinc intake in parts of the population. Only one of 25 studies in vegetarians from the Netherlands and Belgium [57] and three out of 13 studies in vegans from the UK, USA and Australia [25,29,58] reported mean zinc intakes above the EAR. 

Status: Seven studies reported on zinc status, four excluding supplement users. Across studies, average zinc status tended to be slightly lower for vegetarians (0.81 mg/L) and vegans (0.79 mg/L) than for meat-eaters (0.90 mg/L). This was more pronounced in studies excluding supplement users. Individual studies that directly compared zinc status between dietary patterns, showed that compared to meat-eaters, vegetarians had mainly similar (3/6 studies) or significantly lower (2/6 studies) zinc levels. Two out of three studies also found significant lower zinc status in vegans compared to meat-eaters. Three studies reported on zinc deficiency (serum fasting zinc <74/70 ug/dL in men/women). Across studies, the average prevalence of zinc deficiency was similar in vegetarians (14%) and meat eaters (13%), but considerably higher in vegans (30%).

## 4. Discussion

### 4.1. Main Findings and Their Significance

This systematic review provides a comprehensive overview on nutrient intake and status of adults following a predominantly plant-based diet as compared to those following a dietary pattern containing meat. The outcomes are summarized in Table 2.

For energy and macronutrients, we found that energy intake was similar across dietary patterns. Compared to meat-eaters, average protein, EPA and DHA intake was lower in vegetarians and particularly vegans, yet intake of fiber, PUFA, total n-3 fatty acids and ALA was higher in plant-based dietary patterns. Except for EPA and DHA, mean intake of energy and macronutrients of plant-based diets was within the recommendations. In meat-eaters, mean intake of fiber, PUFA and ALA were below recommendations

For micronutrients, vegetarians and vegans generally had lower vitamin B12, vitamin D and iodine intake and status and higher rates of bone turnover markers compared to meat-eaters. Mean iron and zinc intakes were inadequate in vegetarians and vegans due to higher requirements because of lower bioavailability of these micronutrients in plant-based diets. On the other hand, compared to meat-eaters, folate, vitamin E and magnesium intakes were higher in vegetarians and vegans, and vitamin B1, B6 and C intakes were especially higher in vegans. In meat-eaters, mean intake of vitamin E and D was inadequate. Mean calcium intakes were slightly above the EAR for all dietary patterns. Furthermore, mean intakes of vitamin A, B2, niacin and phosphorus were adequate and similar among all dietary patterns.

Our findings imply that plant-based dietary patterns can increase the risk of inadequate intake and status of certain nutrients, which are mainly present or more bioavailable in animal foods (EPA/DHA, vitamin B12, D, iodine, iron, zinc, calcium), but can improve the intake of other nutrients, which are abundant in plant foods. Conversely, meat-eaters are more at risk of inadequate intake of nutrients that are more present in plant foods (fiber, PUFA, ALA, vitamin E, folate, magnesium). However, the inadequate fiber, vitamin E and magnesium intake in more than 25% of the studies in vegetarians indicates that intake of plant-foods may be suboptimal in some populations. Public health strategies are needed to provide guidance and facilitate behavior change to help populations transition to a nutritionally balanced plant-based diet.

### 4.2. Strenghts and Limitations of This Review

The current review is the first systematic review aiming to quantify the differences in nutrient intake and status among different dietary patterns in adults, including meat-eaters, vegetarians, and vegans. A strength of this literature review was that we applied common definitions for the different dietary patterns across all studies to ensure a consistent interpretation of the data. Likewise, for comparability and consistency, we only included studies that used biomarkers and cut-off levels as applied by the IOM and WHO to assess nutritional status and deficiencies. The restriction of the studies to the years 2000–2020 increases the possibility of data being reflective of the current situation, thereby increasing the validity of this study. The findings of our review may help public health authorities and policy makers to develop practical guidance to consumers to help them transitioning to more healthy and sustainable diets, which should consist of a variety of nutrient-dense plant foods.

A major limitation of our review was that we could not provide reliable estimations on the adequacy of dietary nutrient intakes. The prevalence of inadequacy depends on the shape and variation of the usual intake distribution. Therefore, to evaluate the adequacy of dietary intake in a population, ideally the proportion of the population with usual intakes below the EAR should be determined [59]. However, most studies did not provide this information. Instead, we compared mean or median intakes with the EAR (or the lower bound of the AMDR) to indicate (in)adequacy of nutrient intakes in the population. It can be assumed that if mean intake is at or below this level, a substantial proportion of the population will have intakes less than the requirement and is therefore at risk of deficiency. For ALA and fiber, for which only an Adequate Intake (AI) level is available (i.e., based on nutrient intake of a group of healthy people, who are assumed to be adequate), it is not possible to make any assumptions about the prevalence of inadequacy if mean intakes are below the AI, because actual requirements for these nutrients are unknown. Only, when mean intake is above the AI, prevalence of inadequate intakes can be assumed to be low [59].

Another limitation of this review is that the vast majority of studies was conducted in developed, Western countries, mostly in Europe or North America. An exception was the evaluation of vitamin B12, for which a larger part of studies was conducted in Asia. Therefore, our results are mostly applicable to Western populations. The lack of studies from low- and middle-income countries (in particular from Africa) may be explained by the exclusion of studies that assessed habitual, monotonous plant-based diets from populations living in poverty and food insecure situations. These diets are largely consisting of staple foods and lack nutrient-rich plant foods, while food intake is generally limited and therefore the risk of nutrient inadequacies is high for all nutrients [2].

Lastly, we did not detect major differences between studies that assessed intake from foods only or from foods and supplements (except for vitamin B12 and D). This may be explained by heterogeneity in the proportion of supplement users across studies, as well as dose and type of vitamin and minerals used. Moreover, from the included studies it was not clear whether intake from fortified foods was considered, which may have led to an underestimation of micronutrient intakes.

### 4.3. Findings on Energy and Macronutrients

Whereas some studies have suggested that energy intake in people consuming plant-based diets is lower compared to that of meat-eaters [32,33,43], our review showed that mean energy intake was similar and adequate among different dietary patterns.

Despite a somewhat lower average protein intake in the plant-based dietary patterns, all studies reported protein intakes within the level of the adequate macronutrient distribution range. Because the overall protein quality of a vegan or vegetarian diet is estimated to be about 80% and 90% compared to the diet of meat-eaters (i.e., mainly due to the lower digestibility of plant proteins) [60,61], it has been suggested that dietary protein requirements of vegetarians and vegans should be increased by about 20% [61,62]. The mean protein intakes found in our review would still exceed such a potentially increased requirement (i.e., 12% E instead of 10% E). However, WHO and IOM do not specify increased protein requirements for vegetarians and vegans because diverse plant-based diets consisting of different plant proteins with complementary amino acid profiles can provide all essential amino acids and the lower digestibility of plant proteins can be improved through processing and preparation methods [60,63]. Nevertheless, for older adults it can be difficult to obtain sufficient protein from plant-based diets, due to increased protein needs and reduced overall food intake [64,65].

The higher intakes of fiber, PUFA, total n-3 fatty acids (mainly ALA) in plant-based dietary patterns (with the highest intakes in vegan diets) can be explained by the higher intake of plant foods in general, which are rich in these nutrients. While PUFA, n-3 fatty acid and ALA intakes were above the lower AMDR, in about one-third of the studies in vegetarians, fiber intake was below the recommendations, suggesting that not all vegetarians consume sufficient plant foods. However, EPA and DHA intake of vegetarians and vegans was far below the lower AMDR (i.e., 250 mg/d EPA and DHA) because of the absence of fish and seafood from the diet. While eggs can supply some EPA and DHA [66], algae are the only direct plant source of long-chain n-3 fatty acids, but are usually only consumed in small amounts [67,68]. Furthermore, EPA and DHA can also be synthesized in the body from ALA, however, the capacity for conversion remains generally limited (i.e., <10%) [66]. The latter is confirmed by consistent observations of lower EPA and DHA status in vegans and vegetarians compared to meat-eaters and may suggest the need for supplements or fortified foods. Conversely to vegetarians and vegans, meat-eaters’ EPA and DHA intake was in line with the AMDR, but their average fiber, PUFA and ALA intakes failed to meet the lower AMDR or AI. Therefore, meat-eaters would benefit from higher intakes of plant foods such as whole grain products, pulses, nuts, seeds, and some plant oils.

### 4.4. Findings on Micronutrients

Our review showed that intake and/or status of vitamin B12, D, calcium, iron, and zinc may not be sufficient in plant-based diets as these are low, lacking or have a low bioavailability in plant foods.

Most studies in vegans and half of the studies in vegetarians indicated that vitamin B12 intake was inadequate, which was confirmed by a high mean prevalence of vitamin B12 deficiency among vegans (44%) and vegetarians (32%). Our findings are in line with those of earlier reviews [10,12,69]. Vitamin B12 is only present in animal foods, while fermented soy products (e.g., miso, tempeh), shiitake mushrooms, algae and unfortified nutritional yeast contain analogues of vitamin B12, which have been reported to be inactive and may even block the absorption of true vitamin B12 when intake is low [70]. For an adequate vitamin B12 supply, vegans rely on regular use of fortified foods or supplements. Vegetarians can obtain vitamin B12 from dairy and eggs but may also benefit from fortified foods or supplemental vitamin B12 when intake of animal-based foods is limited.

Vitamin D is naturally present only in a few foods, particularly fatty fish, eggs, meat, mushrooms treated with UV-light and algae [71], which explains why highest vitamin D intakes were seen in pesco-vegetarians with regular fish consumption. However, mean vitamin D intakes (also when intake from supplements was considered) were far below the EAR in all dietary patterns and therefore vitamin D fortified foods or supplements are generally recommended [72]. While vitamin D can also be synthesized in the skin upon exposure to sunlight, this may be insufficient during winter in areas of higher latitude (around > 40 °N, e.g., Madrid, Beijing) and for people with dark skin or limited sun exposure [72], as illustrated by the high prevalence of vitamin D deficiency in a small study among Finnish vegans and vegetarians (67% and 33%) [46].

Calcium intake was lower in vegans compared to vegetarians and meat-eaters. However, for all dietary patterns, one third or more of studies reported intakes below the EAR, suggesting that inadequate intakes may occur in the general population. While dairy foods are important sources of calcium, green leafy vegetables, beans, pulses, seeds, nuts and grains are plant foods that are high in calcium, too [70]. It is important to note that calcium absorption may be reduced by phytates and oxalates from plant foods, as well as by insufficient dietary protein intake and low vitamin D status [70]. When habitual calcium intakes are low, calcium absorption is upregulated [70] and PTH production is increased, which stimulates tubular calcium reabsorption and bone resorption [73]. Our review found significantly higher levels of PTH and bone turnover markers in vegans and vegetarians and lower BMD of the lumbar spine in vegans, suggesting greater bone resorption as compared to meat-eaters. Our findings are in line with two recent systematic reviews and meta-analysis, which found that vegetarians and vegans had lower lumbar spine, femoral neck, and whole-body BMDs than omnivores [74,75]. Bone formation and resorption is not only influenced by calcium intake, but also other nutrients such as vitamin D, magnesium, and protein. Therefore, biomarkers on bone health are no direct measure for calcium status, but they can be used to evaluate the overall effect of nutritional intake of different dietary patterns on bone metabolism [73].

Despite similar or higher intakes of iron in vegetarians and vegans as compared to meat-eaters, vegetarians and vegans, particularly women, had lower iron status than meat-eaters, and higher prevalence of iron deficiency and anemia. These findings have been confirmed in previous reviews [9,76] and can be explained by the fact that iron bioavailability in a plant-based diet is substantially lower (i.e., ~10%) compared to a diet with meat and fish containing heme iron (18%) [19]. Heme iron is more efficiently absorbed (15–40%) than non-heme iron (1–15%) [19] which is inhibited by phytates, polyphenols and proteins from milk and eggs [77]. Because of the lower bioavailability of iron from plant-based diets, IOM estimates that dietary requirement of iron is 1.8 times higher for vegetarians and vegans [19]. Notably, vegetarians had a lower mean iron intake than vegans. A possible explanation could be that dairy, unlike protein-rich plant foods, does not contain iron. Therefore, vegetarians who use dairy foods as their major protein source, should be educated to include other iron rich foods in their diet [71]. In addition, absorption of iron from plant-foods can be improved by consumption of vitamin C rich fruits and vegetables [77].

Like iron, zinc absorption from plant-based diets can be reduced due to the higher amount of phytate and fiber. Therefore, dietary requirements for vegetarians and vegans can be increased by up to 50% in high phytate diets as suggested by IOM [14,19]. While mean zinc intakes were similar across dietary patterns, vegans and vegetarians failed to meet the bioavailability-adjusted EAR which was confirmed by lower zinc status and higher prevalence of deficiency, particularly in vegans. Our findings are in line with those of earlier reviews and meta-analysis showing a lower zinc intake and status in vegetarians and vegans, particularly in women, compared to meat-eaters [14,78]. However, there are no reported adverse health consequences in adult vegetarians with lower zinc status, suggesting that the efficiency of zinc utilization may be increased in vegetarians on the longer term [14].

The few studies reporting on iodine indicated a lower intake and status in plant-based diets compared to meat-eaters. Iodine content of animal-based foods is usually higher than in plant-based foods, with fish and dairy as richest sources of iodine [79]. Marine algae are a very concentrated source of iodine, but their iodine content can vary a lot and consumption can lead to excessive intake [80,81]. Because of the limited number of foods with iodine, consumption of iodized table salt and foods produced with iodized salt (e.g., bread, bouillon cubes and seasonings) is recommended by WHO regardless of dietary pattern [82].

We also found that plant-based as compared to meat-containing dietary patterns provided a better supply of some micronutrients, including folate, vitamin E and magnesium, with highest intakes in vegans. Mean intake of folate and magnesium was adequate in vegans and vegetarians, while in 38–57% of the studies meat-eaters failed to meet the requirements. For vitamin E, vegans had an adequate intake, but 43% and 64% of the studies showed inadequate intakes in vegetarians and meta-eaters, respectively. Vitamin E intake in the diet often corresponds with PUFA intake. However, due to higher PUFA intakes, more vitamin E is needed to protect PUFA in cell membranes and plasma lipoproteins from oxidation by free radicals [83]. This may explain why a higher vitamin E intake in vegans was not reflected in a higher vitamin E status.

The intake of vitamin B1,B6 and C was higher in vegans, yet intakes were generally above the EAR across all dietary patterns. Good sources of vitamin B1 and B6 are wholegrains, pulses, nuts and seeds but also meat and fish [19,83], while fruits and vegetables are the major sources of vitamin C.

For vitamin A, B2, niacin and phosphorus, mean intakes were well above the EAR and similar across dietary patterns. For vitamin A, intakes may have been overestimated for plant-based diets as factors to convert carotenoids to retinol were not described in all studies. A study from the UK that estimated inadequate intakes of total vitamin A based on different conversion factors, showed that the prevalence of inadequate vitamin A intake increased from 3–8% when using 1:6 factor for beta-carotene conversion (RE) to 9–22% in vegetarians and 20–37% in vegans when using 1:12 factor for beta-carotene conversion (RAE) [84]. While the limited data in our review did not show any presence of vitamin A deficiency in vegetarians and vegans, more research with recent validated biomarkers of vitamin A status may help to conclude which conversion factor will be relevant.

### 4.5. Implications for Public Health and Recommendations

A shift to a diet with more plant foods and less animal foods can improve the intake of fiber, PUFA, folate, vitamin B1, B6, C, E, and magnesium and subsequently benefit health outcomes. In particular, intake of fiber, PUFA, folate and vitamin E was found to be inadequate in meat-eaters compared to vegetarians and vegans in the current review.

On the other hand, careful planning is needed to consume a nutritious plant-based diet as there is a risk of inadequate intakes of EPA, DHA, vitamin B12, D, calcium, iron, zinc, and iodine (of which vitamin D and calcium are also of concern in meat-eaters). In addition, in the case of vegetarian diets, intakes of fiber and vitamin E may be insufficient when foods rich in these nutrients are not consumed in adequate amounts.

Therefore, in the transition to more healthy plant-based diets, health authorities will need to educate consumers to adopt a diverse diet with foods rich in these nutrients and facilitate behavior change. In addition, for vitamin B12, which is absent in plant foods, and for vitamin D and iodine, which can naturally only be found in a limited number of foods, additional public health strategies are needed, including food fortification and universal salt iodization. For iron, zinc and possibly calcium and provitamin A carotenoids, guidance should include advice to improve the bioavailability and bioconversion of these nutrients to avoid deficiencies (e.g., to consume vitamin C rich foods with meals to improve iron absorption). Industry can play a role in designing nutritious products and recipes that can help to increase the nutrient intake and bioavailability of these nutrients. Supplementation can be an alternative strategy, and our review showed that mean vitamin B12 intake and status was higher and adequate in studies that considered supplement use. However, for vitamin D, intakes in most studies remained inadequate, even when intake from supplements was considered. Additionally, mean intakes of other micronutrients did hardly differ between studies that assessed intake from foods only or from foods and supplements. This may be explained by heterogeneity in use, dose, and type of micronutrients among studies. Therefore, supplement use may not always be the preferred strategy to improve micronutrient intake as they are possibly used by a select group of people, who are health conscious and can afford them.

## 5. Conclusions

We conclude that there are dietary inadequacies in all dietary groups. In people following self-selected plant-based diets, especially vegan diets, intake, and status of certain nutrients is lower compared to meat-containing diets, with an increased risk of inadequacy for vitamin B12, vitamin D, EPA, DHA, calcium, iron (particularly in women), zinc and iodine. Of these nutrients, also meat-eaters were found to be at risk of inadequate vitamin D and calcium intake. On the other hand, people following plant-based diets, particularly vegan diets, had higher intakes of PUFA, ALA, fiber, folate, vitamin E and magnesium, which were found to be at risk of inadequacy among meat-eaters. Additionally, the intake of vitamin B1, B6 and C was considerably higher, especially in vegans.

Our results show the need for additional public health strategies to help consumers transitioning to a more nutritionally balanced and sustainable diet by education on diverse nutrient-dense plant foods, food fortification and possibly supplementation.

## Figures and Tables

**Table 1:** Characteristics of the 141 included studies in adults.

Characteristics	Number of Studies (n)
Europe	74 (mostly Germany, UK)
South/East Asia	33 (mostly Taiwan, India, China)
North America	22 (mostly US)
Australasia	8
South America	2 (Brazil)
West Asia	2 (Israel, Jordan)
Women only	27
Men only	9
Older adults ^1^	11
Nutrient intake, assessed from foods only	66
Nutrient intake, assessed from foods and supplements	17
Nutrient status in non-users of supplements	44
Nutrient status in users and non-users of supplements	55
Meat-eating	101
Vegetarian	118
Vegan	63
Semi-vegetarian	7
Pesco-vegetarian	6
High/medium/low animal protein intake	2
High/medium/low meat intake	1

^1^ Post-menopausal women (mean age 52–60 year) and older men/women (mean age 62–84 year).

**Table 2:** Overview of nutrients at risk of inadequacy and nutrients of favorably high intake across dietary patterns.

Dietary Pattern	Nutrients at Risk of Inadequacy	Nutrients of Favorably High Intake
Vegans	EPA, DHA,	fiber, PUFA, ALA,
	vitamins B12, D,	vitamins B1, B6, C, E, folate,
	calcium, iodine, iron (in women), zinc	magnesium
Vegetarians	fiber, EPA, DHA,	PUFA, ALA,
	vitamins B12, D, E,	vitamin C, folate,
	calcium, iodine, iron (in women), zinc	magnesium
Meat-eaters	fiber, PUFA, ALA (in men),	protein,
	vitamins D, E, folate,	niacin, vitamin B12,
	calcium, magnesium	zinc

## Data Availability

Data is contained within the article or Supplementary Material.

## Supplementary Materials

The following are available online at https://www.mdpi.com/article/10.3390/nu14010029/s1, Figure S1, Nutrient intake from studies assessing intake from food only and from studies assessing intake from foods and supplements, Table S1: Details of individual studies, Table S2: Nutritional status, hemoglobin, and bone marker data per dietary pattern.
