# Supplementary material for: Nutrient Intake and Status in Adults Consuming Plant-Based Diets Compared to Meat-Eaters: A Systematic Review
Source: Nutrients. 2021 Dec 23;14(1):29. doi: 10.3390/nu14010029 (PMC8746448; doi:10.3390/nu14010029)
Supplement: Supplementary file 1 [file nutrients-14-00029-s001.zip › Supplementary figure S1_intake from foods vs foods & supplements.pdf]

Supplementary material Figure S1: Nutrient intake from studies assessing intake from food only and from studies assessing intake from foods and supplements

A) Vegetarians

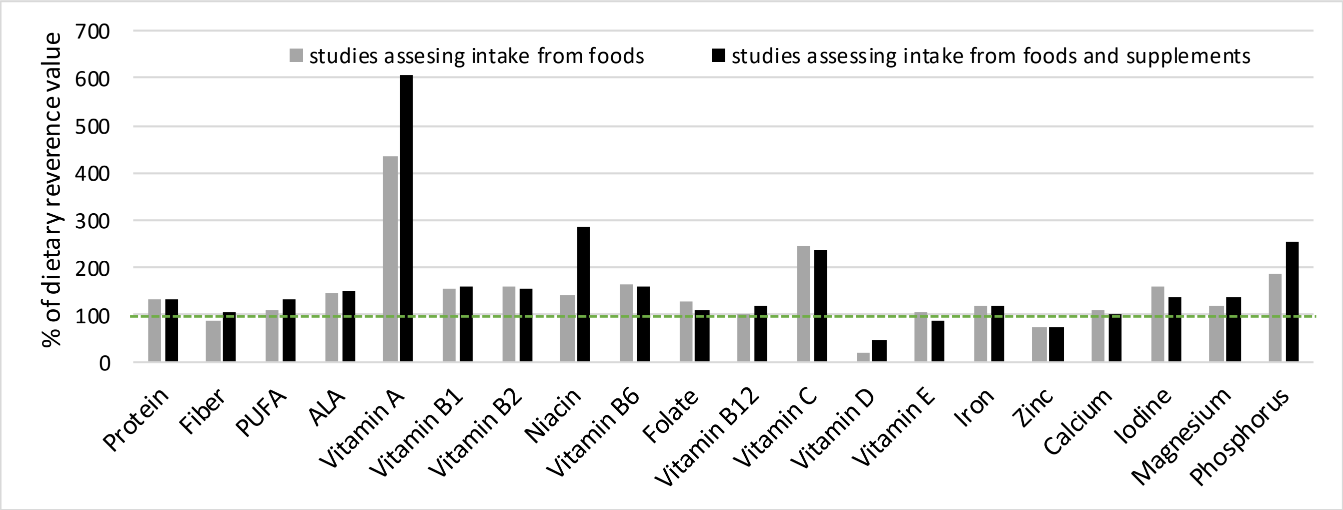

B) Vegans

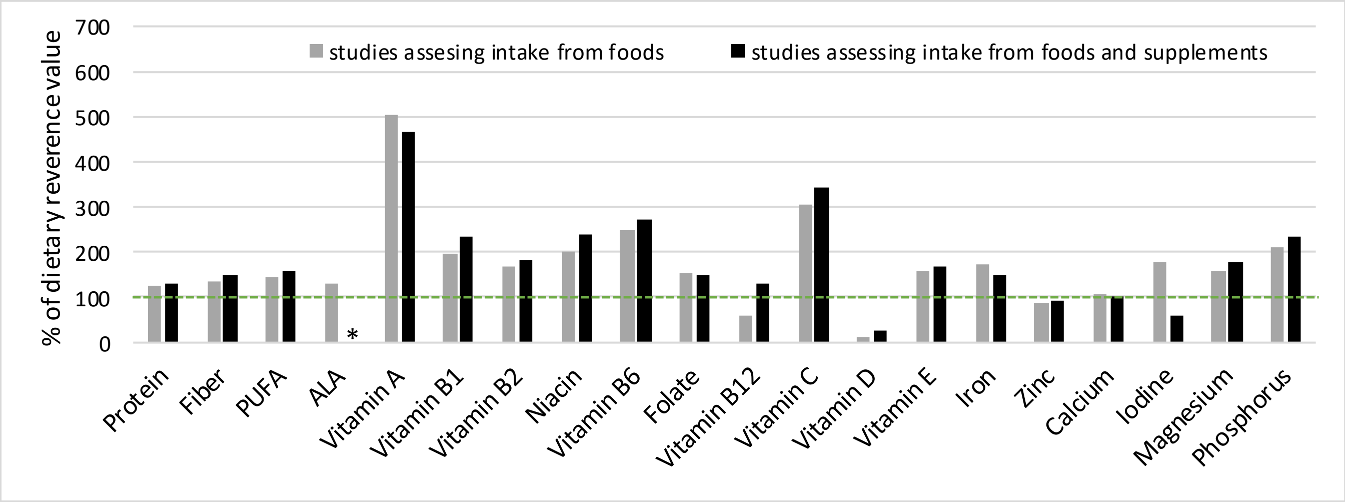

C) Meat-eaters

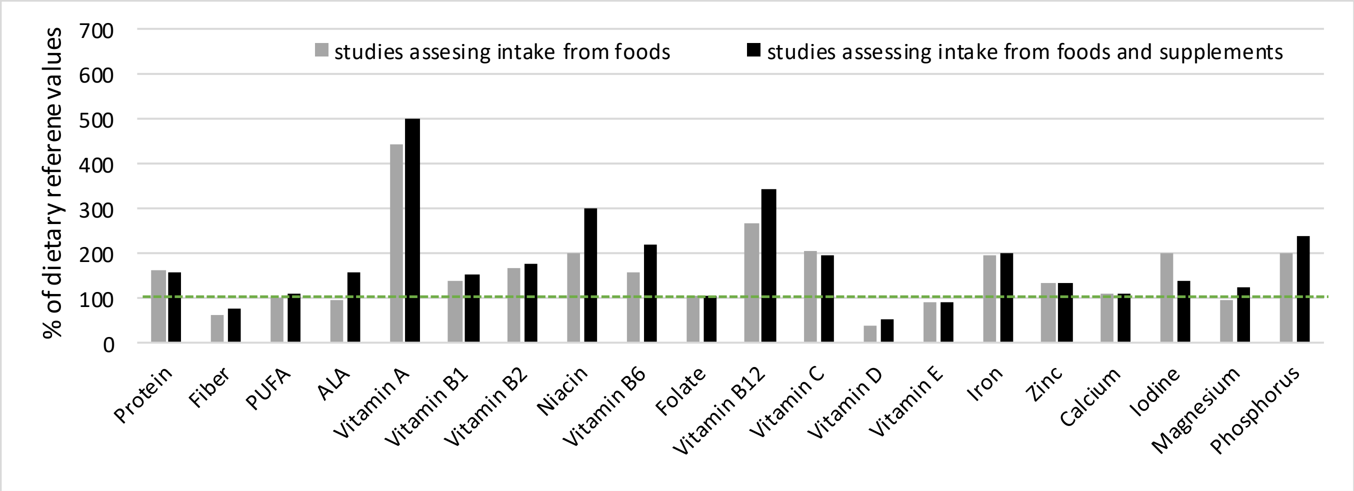

Dietary referene values: for protein and polyunsaturated fatty acids (PUFA) the lower level of the acceptable macronutrient distribution range; for fiber and α-linolenic acid (ALA) the Adequate Intake. For all vitamins and minerals the estimated average requirement (EAR). For vegetarians and vegans, the EAR for iron and zinc was adjusted for lower bioavailability from plant-based diets. If dietary reference values differed for men and women, the average value was used.

* No studies assessing ALA intake from foods & supplements in vegans were available
