# Supplementary material for: Nutrient Intake and Status in Adults Consuming Plant-Based Diets Compared to Meat-Eaters: A Systematic Review
Source: Nutrients. 2021 Dec 23;14(1):29. doi: 10.3390/nu14010029 (PMC8746448; doi:10.3390/nu14010029)
Supplement: Supplementary file 1 [file nutrients-14-00029-s001.zip › Supplementary table S1_Study details.pdf]

Table S1. **Details of individual studies**

Reference	Country	Study population	Dietary pattern	n	Age (y)	Age_unit	Age variation	Age_var_ unit	Reported data	Supplements	Dietary assessment method
Al Delaimy 2004	UK	adults	vegetarians	198	54.9	mean	5.8	SD	status: vit B1, B2, B6, folate, B12.	users & non-users of supplements	n.a.
Allen 2008	USA	adults	vegetarians	not reported	not reported	not reported	19-50	range	status: vit B12	users & non-users of supplements	n.a.
			meat-eaters	not reported	not reported	not reported	19-50	range			
Alles 2017	France	adults	vegetarians	2370	48.7	mean	14.7	SD	intake: energy, protein, fiber, PUFA, total n-3 FA, vit A, B1, B2, B6, B12, niacin, folate, C, D, E, iron, zinc, calcium, iodine, magnesium, phosphorus	foods & supplements	24h recall (three times)
			vegans	789	48.7	mean	14.7	SD			
			meat-eaters	90664	48.7	mean	14.7	SD			
Andrich 2011	USA	women	vegetarians	22	43	mean	16	SD	intake: energy, protein, fiber, PUFA	foods only	3d food record (twice)
			vegans	20	36	mean	14	SD			
			meat-eaters	21	40	mean	15	SD			
Ball 2000	Australia	women	vegetarians	50	25.2	mean	18-45	range	intake: energy, protein, fiber, total n-3 FA, EPA, DHA, iron, zinc	foods & supplements	4d weighted record (3 times)
			meat-eaters	24	25.3	mean	18-45	range	status: zinc	users & non-users of supplements	
		men	vegetarians	39	not reported	not reported	20-50	range			
			meat-eaters	25	not reported	not reported	20-50	range			
			vegans	10	not reported	not reported	20-50	range			
Barghouti 2009	Jordan	adults	vegans	53	not reported	not reported	18-78	range	status: vit B12	non-users of supplements	n.a.
			vegetarians	163	not reported	not reported	18-78	range			
			meat-eaters	785	not reported	not reported	18-78	range			
Bedford 2005	Canada	adults	vegetarians	106	41.5	mean	1.8	SD	intake: energy, protein, fiber, vit A, B1, B2, B6, B12, niacin, folate, C, iron, zinc, calcium, magnesium, phosphorus	foods & supplements	24h recall
			meat-eaters	1711	44.8	mean	0.4	SD			
Beezhold 2010	USA	adults (Adventists)	vegetarians	60	45.07	mean	1.42	SD	intake: total n-3 FA, ALA, EPA, DHA	foods only	FFQ
			meat-eaters	78	41.00	mean	1.4	SD			
Bhatti 2007	India	adults	vegetarians	136	32.9	mean	11.3	SD	status: iron	users & non-users of supplements	n.a.
			meat-eaters	150	33.8	mean	11.3	SD			
Bissoli 2002	Italy	general population	vegetarians	14	48.5	mean	14.5	SD	intake: energy, protein, fiber, vit B6, folate	foods only	not reported
			vegans	31	45.8	mean	15.8	SD	status: folate, vit B12	non-users of supplements	
			meat-eaters	29	43.4	mean	16.7	SD			
Boanca 2014	Romania	adults	vegetarians	48	28.4	mean	8.6	SD	status: vit B12	non-users of supplements	n.a.
			meat-eaters	38	29.8	mean	10.1	SD			
Brantsæter 2018	Norway	adults	vegetarians	25	37.2	mean	15.7	SD	intake: iodine	foods & supplements	24h recall (twice)
			vegans	19	30.1	mean	7.1	SD	status: iodine	users & non-users of supplements	
			meat-eaters	71	42.1	mean	11.4	SD			
Burns-Whitmore 2010	USA	adults	vegetarians	20	38	mean	3	SD	status: PUFA, total n-3 FA, ALA, EPA, DHA	non-users of supplements	n.a.
Burns-Whitmore 2014	USA	adults	vegetarians	20	38	mean	3	SD	status: PUFA, total n-3 FA, ALA, EPA, DHA	non-users of supplements	n.a.
Cade 2004	UK	women	vegetarians	10634	49	mean	9	SD	intake: energy, protein, fiber, PUFA, vit A, folate, C, iron, zinc, calcium	foods only	FFQ
			meat-eaters	24738	54	mean	9	SD			
Carmel 2002	USA	adults (Asian Indian immigrants)	vegetarians	8	29.9	mean	20-69	range	status: folate, vit B12, D	users & non-users of supplements	n.a.
Chan 2009	USA	adults	vegetarians	150	61.6	mean	13.3	SD	intake: vit D	foods & supplements	FFQ
			semi-vegetarians	66	61.3	mean	13.3	SD	status: vit D	users & non-users of supplements	
			meat-eaters	212	60.2	mean	13.3	SD			
Chang 2019	Taiwan	adults	vegetarians	1403	54.0	mean	9.3	SD	intake: energy, protein	foods & supplements	FFQ

Sensitivity: Confidential

			meat-eaters	3436	51.8	mean	10.5	SD			
Chiu 2014	Taiwan	adults	vegetarians	1484	55.1	mean	7.5	SD	intake: energy, protein, fiber, PUFA, vit B1, B2, B6, B12, niacin, folate, C, iron, zinc, calcium, magnesium	foods only	FFQ
			meat-eaters	2900	53.9	mean	7.9	SD			
Clarys 2013	Belgium	adults	vegetarians	69	35 (m) 39 (w)	mean	12 (m) 14 (w)	SD	intake: energy, protein, PUFA	foods only	3-d food diary
			meat-eaters	69	36 (m) 40 (w)	mean	12 (m) 14 (w)	SD			
Clarys 2014	Belgium	adults	vegans	104	not reported	not reported	20-69	range	intake: energy, protein, PUFA, iron, calcium	foods only	52-item qualitative FFQ
			vegetarians	573	not reported	not reported	20-69	range			
			semi-vegetarians	498	not reported	not reported	20-69	range			
			pesco-vegetarians	145	not reported	not reported	20-69	range			
			meat-eaters	155	not reported	not reported	20-69	range			
Crowe 2011	UK	adults	vegetarians	417	44.0	mean	14	SD	intake: energy, protein, vit D, calcium	foods & supplements	FFQ
			vegans	87	48.0	mean	13	SD	status: vit D	users & non-users of supplements	
			pesco-vegetarians	208	51.0	mean	12	SD			
			meat-eaters	1359	55.0	mean	10	SD			
Damayanti 2018	USA	adults (Adventists)	vegetarians	207	not reported	not reported	29-94y	range	intake: vit B12	foods & supplements	24h recall (6 times)
			vegans	67	not reported	not reported	29-94y	range	status: vit B12	users & non-users of supplements	
			pesco-vegetarians	78	not reported	not reported	29-94y	range			
			semi-vegetarians	35	not reported	not reported	29-94y	range			
			meat-eaters	341	not reported	not reported	29-94y	range			
Davey 2003	UK	adults	vegetarians	17860	35.9	mean	20-97	range		foods only	FFQ
			vegans	2396	33.1	mean	20-97	range	intake: energy, protein, fiber, PUFA, vit B1, B2, B6, B12, niacin, folate, C, D, E, iron, zinc, calcium, magnesium		
			pesco-vegetarians	9423	39.5	mean	20-97	range			
			meat-eaters	32308	48.7	mean	20-97	range			
de Bortoli 2009	Brazil	adults	vegetarians	30	27	mean	not reported	not reported	status: zinc	non-users of supplements	n.a.
Deriemaeker 2010 a	Belgium	adults	vegetarians	30	23	mean	2	SD	intake: energy, protein, calcium, magnesium, phosphorus	foods only	3-day food record
			meat-eaters	30	24	mean	3	SD			
Deriemaeker 2010 b	Belgium	adults	vegetarians	106	36	mean	20-85	range	intake: energy, protein, fiber, PUFA, vit C, iron, zinc, calcium, magensium, phosphorus	foods only	3d food record
			meat-eaters	106	37	mean	20-85	range			
Deriemaeker 2011	Netherlands	adults	vegetarians	29	84.1 (f) 80.5 (m)	mean	5.1 (f) 7.5 (m)	SD	intake: energy, protein, fiber, PUFA, vit A, B1, B2, C, D, iron, zinc, calcium, magnesium, phosphorus	foods only	FFQ
			meat-eaters	30	84.3 (f) 80.6 (m)	mean	5.0 (f) 7.3 (m)	SD	status: folate, vit B12, iron zinc	users & non-users of supplements	
Dyett 2013& 2014	USA	adults	vegans	100	25-75	range	n.a.	n.a.	intake: vit B12, D, iron, zinc, calcium	foods only	FFQ
Elorinne 2016	Finland	adults	vegans	22	33	mean	24-50	range	intake: energy, protein, fiber, PUFA, vit A, B1, B2, B6, B12, niacin, folate, C, D, E, iron, zinc, calcium	foods only	3-d food record
			meat-eaters	19	35	mean	24-52	range	status: folate, vit B12, D, iron, zinc, iodine, ALA, EPA, DHA	users & non-users of supplements	
Farmer 2011	USA	adults	vegetarians	851	≥19	range	n.a.	n.a.	intake: energy, protein, fiber, vit A, B1, B2, B6, B12, niacin, folate, C, E, iron, zinc, calcium, magnesium	foods only	24h recall
			meat-eaters	12441	≥19	range	n.a.	n.a.			
Fayet 2014	Australia	young women	vegetarians	23	22.6	mean	3.9	SD	intake: energy, protein, total n-3 FA, vit B12, iron, zinc	foods only	FFQ
			meat-eaters	200	22.6	mean	3.9	SD			
Fokkema 2000a	Netherlands	adults	vegans	12	38.0	mean	12.5	SD	intake: PUFA	foods only	FFQ
			meat-eaters	15	35.2	mean	8.9	SD	status: PUFA, total n-3 FA, ALA, EPA, DHA	users & non-users of supplements	

Fokkema 2000b	Netherlands	adults	vegans	12	41	mean	12.0	SD	intake: PUFA, ALA	foods only	FFQ
Fokkema 2005	Netherlands	adults	vegetarians	20	40.2	mean	14.0	SD	status: ALA, EPA, DHA	users & non-users of supplements	n.a.
Gallego-Narbón 2018	Spain	adults	vegetarians	49	31.2	mean	8.3	SD	status: vit B12	users & non-users of supplements	n.a.
			vegans	54	29.5	mean	7.1	SD			
Gallego-Narbón 2019 a & b	Spain	adults	vegetarians	49	31.3	mean	8.6	SD	status: folate, vit B12, iron	users & non-users of supplements	n.a.
			vegans	55	29.5	mean	7.1	SD			
Gammon 2012	New Zealand	overweight women (Asian immigrants)	vegetarians	34	36.6	mean	10.1	SD	status: folate, vit B12	non-users of supplements	n.a.
			meat-eaters	90	40.3	mean	9.08	SD			
Geisel 2005	Germany	adults	vegetarians	48	53	median	21, 75	25th, 75th percentiles	status: vit B6, folate, vit B12	users & non-users of supplements	n.a.
			vegans	23	51	median	28, 76	25th, 75th percentiles			
			meat-eaters	79	51	median	23, 68	25th, 75th percentiles			
Geppert 2005	Germany	adults	vegetarians	114	not reported	not reported	18-43	range	intake: energy, protein, fiber, EPA, DHA	foods only	3d food record
									status: ALA, EPA, DHA	non-users of supplements	
Geppert 2006	Germany	adults	vegetarians	106	25.9	mean	5.6	SD	status: vit E, EPA, DHA	non-users of supplements	n.a.
Gilsing 2010	UK	men (all)	vegetarians	231	46.2	mean	11.7	SD	intake: energy, protein, vit B12, folate	foods & supplements	FFQ
			vegans	232	42.8	mean	13.1	SD	status: folate, vit B12	users & non-users of supplements	
			meat-eaters	226	52.8	mean	10.7	SD			
		men (non-supplement users only)	vegetarians	186	not reported	not reported	not reported	not reported	intake: energy, protein, vit B12, folate	foods only	FFQ
			vegans	189	not reported	not reported	not reported	not reported			
			meat-eaters	216	not reported	not reported	not reported	not reported			
Goff 2005	UK	adults	vegans	21	35	median	23–69	range	intake: energy, protein, fiber, PUFA	foods only	7d food record
			meat-eaters	25	36	median	24–75	range			
Gojda 2013	Czech republic	adults	vegans	11	29.47	median	25.8–36.8	range	intake: energy, protein	foods only	3d food record
			meat-eaters	10	27.23	median	25.6–28.9	range	status: PUFA, ALA, DHA	users & non-users of supplements	
Gómez-Donoso 2019	Spain	adults	high meat-eaters	2939	35	mean	11	SD		foods only	FFQ
			moderate meat-eaters	1781	35	mean	11	SD	intake: energy, protein, fiber, PUFA, folate, vit C, D, calcium, magnesium		
			low meat-eaters	2245	35	mean	11	SD			
Gupta 2004	Canada	adults	vegetarians	172	not reported	not reported	18 - 84	range	status: vit B12	users & non-users of supplements	n.a.
			meat-eaters	23	not reported	not reported	19 - 84	range			
Haddad 2003	USA	general population	vegetarians	120	not reported	not reported	6-70+	range	intake: protein, fiber, PUFA, vit A, B1, B2, B6, B12, niacin, folate, C, E, iron, zinc, calcium, magnesium, phosphorus	foods only	24h recall (twice)
			meat-eaters	12543	not reported	not reported	6-70+	range			
Hansen 2018	Denmark	adults	vegans	78	31	mean	8	SD	intake: energy, calcium	foods only	4d weighed food record
			meat-eaters	77	32	mean	8	SD	status: vit D, calcium, bone markers, magnesium	non-users of supplements	
Haroon 2012	India	adults	vegetarians	116	26	mean	5.0	SD	intake: vit B12	foods only	FFQ
			meat-eaters	35	26	mean	5.0	SD	status: folate, vit B12	non-users of supplements	
Harvey 2005	UK	women	vegetarians	30	31	mean	6.0	SD	intake: iron	foods only	7-d dupliate diet collection & 7-d food record
			meat-eaters	60	33	mean	6.5	SD	status: iron	non-users of supplements	
Hawk 2012	USA	female university students	vegetarians	19	19.7	mean	1.6	SD	intake: energy, protein, fiber, PUFA, vit C, iron, calcium, phosphorus	foods only	3d food record
			meat-eaters	20	20.7	mean	1.0	SD	status: iron	non-users of supplements	
He 2016	China	adults	low animal protein	580	37.8	mean	8.4	SD	intake: energy, protein, fiber, vit A, C, iron, zinc, calcium, magnesium	foods only	24h recall (three times)

Sensitivity: Confidential

			moderate animal protein	580	37.6	mean	8.6	SD	status: iron	users & non-users of supplements	
			high animal protein	581	36.1	mean	9.2	SD			
Henjum 2018	Norway	women	vegetarians	36	22	median	21, 24	25th, 75th percentiles	status: iodine	users & non-users of supplements	n.a.
			meat-eaters	367	22	median	21, 24	25th, 75th percentiles			
Herrmann 2001	Germany	adults	vegetarians	34	22	median	19.5, 49.3	5th, 95th percentiles	status: folate, vit B12	non-users of supplements	n.a.
			vegans	7	22	median	19, 30	5th, 95th percentiles			
			meat-eaters	63	23	median	not reported	not reported			
Herrmann 2003a	Gemany & NL	adults	vegetarians	66	48	median	24, 75	5th, 95th percentiles	status: vit B6, folate, vit B12	users & non-users of supplements	n.a.
			vegans	29	37	median	15, 64	5th, 95th percentiles			
			meat-eaters	79	51	median	23, 68	5th, 95th percentiles			
Herrmann 2003b	Gemany & NL	adults	vegtarians	111	46	mean	15	SD	status: folate, vit B12	users & non-users of supplements	n.a.
			meat-eaters	93	40	mean	17	SD			
Herrmann 2005	Germany	adults	vegetarians	114	50	median	35, 71	10th, 90th percentiles	status: vit B6, folate, vit B12	users & non-users of supplements	n.a.
			vegans	50	44	median	20, 66	10th, 90th percentiles			
			meat-eaters	109	41	median	24, 61	10th, 90th percentiles			
Herrmann 2009	Germany	men	vegetarians	54	50	mean	38–70 y	range	status: folate, vit B12, D	non-users of supplements	n.a.
			vegans	23	45	mean	31–74 y	range			
			meat-eaters	35	41	mean	30–60	range			
	Oman	men (Asian-Indian immigrants)	vegetarians	19	45	mean	40–54	range	status: folate, vit B12, D, bone markers	non-users of supplements	n.a.
			meat-eaters	54	43	mean	40–52	range			
Ho-Pham 2009 & 2012	Vietnam	elderly women	vegetarians	105	62	mean	10	SD	intake: energy, protein, calcium, phosphorus	foods only	2d food record
			meat-eaters	105	62	mean	10	SD	status: vit D, calcium, bone markers	users & non-users of supplements	
Hua 2001	USA	adults	vegetarians	29	41	mean	37-45	95% CI	status: iron	non-users of supplements	n.a.
			meat-eaters	29	40	mean	36-44	95% CI			
Huang 2003	Taiwan	young adults	vegetarians	37	28.9	mean	5.5	SD	intake: energy, protein, fiber, vit B6, B12	foods only	3d food record
			meat-eaters	32	22.9	mean	1.6	SD	status: vit A, B6, folate, B12	non-users of supplements	
Huang 2011	Taiwan	older adults	vegetarians	41	71.2	median	67, 75	25th, 75th percentiles	intake: energy, protein, fiber, vit B6, B12, calcium, magnesium	foods only	24-h recall
			meat-eaters	1030	71.0	median	68, 75	25th, 75th percentiles			
Huang 2013	China	men	semi-vegetarians	103	40	mean	10	SD	status: folate, vit B12, PUFA, total n-3 FA, ALA, EPA, DHA	users & non-users of supplements	n.a.
			meat-eaters	128	44	mean	8	SD			
Hung 2002	Taiwan	women (Buddhists)	vegetarians	45	not reported	not reported	31-45	range	intake: energy, protein, B1, B2, B6, B12, niacin, folate	foods only	24-h recall & semiquantitative FFQ
			meat-eaters	45	not reported	not reported	31-45	range	status: vit B6	non-users of supplements	
Jin 2018	USA	adults	vegetarians	335	55.6	mean	9.2	SD	intake: energy, protein, fiber, PUFA, total n-3 FA, vit A, B1, B2, B6, B12, niacin, folate, C, E, iron, zinc, calcium	foods only	FFQ
			meat-eaters	557	55.2	mean	9.5	SD			
Kajanachumpol 2011	Thailand	adults	vegans	109	not reported	not reported	30-50	range	status: iron	non-users of supplements	n.a.
			meat-eaters	86	not reported	not reported	30-50	range			
Karabudak 2008	Turkey	young women	vegetarians	26	29	mean	8.84	SD	intake: energy, protein, fiber, PUFA, vit A, B1, B2, B6, B12, niacin, folate, C, E, calcium,	foods only	4d food record (twice)
			meat-eaters	26	27.4	mean	6.58	SD	status: folate, vit B12, C, E	non-users of supplements	
Kazimirova 2006	Slovak Republic	adults	vegetarians	24	40	mean	1.7	SD	status: folate, vit B12	users & non-users of supplements	n.a.
			meat-eaters	24	41.2	mean	2	SD			

Sensitivity: Confidential

Kim 2007	South Korea	postmenopausal women	vegetarians	76	60.7	mean	6.9	SD	intake: energy, protein, fiber, iron, zinc, calcium, magnesium, phophorus	foods only	24h recall (3 times)
			meat-eaters	76	60.8	mean	6.7	SD	status: iron, zinc, calcium, BMD, magnesium, phosphorus	non-users of supplements	
Kim 2012	South Korea	postmenopausal women	vegetarians	59	63	mean	9.3	SD	intake: energy, protein, fiber, iron	foods only	24h recall (3 times)
			meat-eaters	48	62	mean	8.3	SD	status: iron	non-users of supplements	
Kapoor 2017	Pakistan	adults	vegetarians	100	27.7	mean	5.85	SD	status: folate, vit B12	non-users of supplements	n.a.
			meat-eaters	100	28.8	mean	5.7	SD			
Kniskern 2011	USA	women	vegetarians	22	26.2	mean	1.4	SD	intake: energy, protein, fiber	foods only	4-d food record
Knurick 2015 & Johnston 2018	USA	adults	meat-eaters	27	27.2	mean	6.7	SD	intake: energy, protein, fiber, total n-3 FA, vit A, B6, B12, folate, C, E, iron, zinc, calcium, magnesium	foods only	FFQ and 24h recall
			vegetarians	26	31.1	mean	9.1	SD		users & non-users of supplements	
			vegans	28	33.9	mean	8.6	SD	status: vit C, BMD		
Kornsteiner 2008	Austria	adults	vegetarians	25	36.4	mean	15.2	SD	intake: PUFA, total n-3 FA, ALA, EPA, DHA	foods only	24h recall, FFQ
			vegans	37	29.5	mean	11.3	SD	status: BMD, PUFA, ALA	users & non-users of supplements	
			semi-vegetarians	13	31.8	mean	12.5	SD			
			meat-eaters	23	38.5	mean	14.6	SD			
Kotova 2015	Sweden	adults	vegetarians	29	not reported	not reported	21-37	range	status: folate, vit B12	users & non-users of supplements	n.a.
			meat-eaters	27	not reported	not reported	21-37	range			
Krajcovicova-Kudlackova 2000	Slovakia	adults	vegetarians	54	37.3	mean	1.0	SD	intake: energy, PUFA, ALA, vit B12, folate, C, E	foods only	FFQ
			vegans	32	41.5	mean	1.8	SD	status: folate, vit B12, C, E	users & non-users of supplements	
			meat-eaters	59	40.9	mean	0.7	SD			
Krajčovičová-Kudláčková 2003a	Slovakia	adults	vegetarians	31	39.1	mean	1.5	SD	status: iodine	users & non-users of supplements	n.a.
			vegans	15	42.2	mean	2.6	SD			
			meat-eaters	35	43.4	mean	1.4	SD			
Krajcovicova-Kudlackova 2003b	Slovakia	adults	vegetarians	110	37.5	mean	0.3	SD	status: folate, vit B12, C, iron, zinc	non-users of supplements	n.a.
			meat-eaters	101	35	mean	0.4	SD			
Krajcovicova-Kudlackova 2007	Slovakia	adults (normal weight)	vegetarians	52	25.2	mean	0.4	SD	status: vit B6, folate, vit B12, C	non-users of supplements	n.a.
			meat-eaters	52	23.9	mean	0.3	SD			
Krajcovicová-Kudlácková 2011	Slovakia	older adults	meat-eaters	38	63.4	mean	0.5	SD	intake: fiber, ALA, vit B6, B12, folate, C	foods only	FFQ
			vegetarians	45	65.2	mean	0.5	SD	status: vit B6, folate, vit B12, C, E	non-users of supplements	
Krajcovicová-Kudlácková 2013	Slovakia	adults	meat-eaters	73	not reported	not reported	20-63	range	status: folate, vit B12	non-users of supplements	n.a.
			vegetarians	54	not reported	not reported	20-64	range			
Kristensen 2015	Denmark	adults	vegans	70	28	mean	25-34	range	intake: energy, protein, fiber, PUFA, vit A, B1, B2, B6, B12, niacin, folate, C, E, iron, zinc, calcium, iodine, magnesium, phosphorus	foods & supplements	4/7-d food record
			meat-eaters	1257	42	mean	33-52	range			
Krivosikova 2010	Slovakia	women	vegetarians	141	41.9	mean	19.7	SD	status: folate, vit B12, calcium, bonemarkers, phosphorus	non-users of supplements	n.a.
			meat-eaters	131	40.8	mean	19.8	SD			
Kuchta 2016	Poland	adults	vegans	21	28	mean	5	SD	intake: energy, protein, fiber, PUFA, vit A, B1, B6, C	foods only	24h recall (three times)
			meat-eaters	21	28	mean	4	SD			
Kwok 2002	China	older women	vegetarians	119	>55	not reported	not reported	not reported	status: folate, vit B12, iron	users & non-users of supplements	n.a.
Kwok 2004	China	older women	vegetarians	113	77.4	mean	7.6	SD	status: folate, vit B12	users & non-users of supplements	n.a.
Kwok 2012	China	adults	vegetarians	50	45+9	mean	9	SD	intake: energy, protein<br>status: folate, vit B12	foods only	FFQ
Leblanc 2000	France	adults	vegetarians	94	51.70	mean	16.5	SD	intake: energy, protein, vit B12, folate, C, iron,	foods only	5d food record

Sensitivity: Confidential

		adults (Hindu)	vegetarians	34	40.41	mean	11	SD	calcium, magnesium		
Lee 2000	China	adults	vegetarians	60	not reported	not reported	25-55	range	intake: energy, protein	foods only	FFQ
			meat-eaters	194	not reported	not reported	25-70	range	status: PUFA, total n-3 FA, ALA, EPA, DHA	users & non-users of supplements	
Lee 2011	Korea	women (Bhuddist nuns)	vegetarians	54	not reported	not reported	21-44	range	intake: energy, PUFA, protein, folate, vit C	foods only	3d food record
		women (Catholic nuns & college students)	meat-eaters	81	not reported	not reported	18-41	range	status: folate, vit B12, iron	non-users of supplements	
Leonard 2014	Australia	young women	vegetarians	31	not reported	not reported	18-35	range	intake: iron	foods only	FFQ
			meat-eaters	76	not reported	not reported	18-35	range	status: iron	non-users of supplements	
Leung 2011	USA	adults	vegetarians	78	30.8	mean	10.6	SD	status: folate, vit B12, iodine	users & non-users of supplements	n.a.
			vegans	62	36.6	mean	13.2	SD			
Li 2000	Australia	men	vegetarians	43	34.9	mean	9	SD	intake: protein, fiber, PUFA, vit A, C, iron, zinc, calcium	foods only	FFQ
			vegans	18	33	mean	7.7	SD	status: vit A, folate, vit B12, E, iron	non-users of supplements	
			meat-eaters	78	37.4	mean	8.4	SD			
Lightowler 2000a	UK	adults	vegans	26	40.1	mean	16.6	SD	intake: energy, protein, fiber, PUFA, iron, zinc, calcium, magnesium	foods & supplements	4d food record
Lightowler 2000b	UK	adults (all)	vegans	26	40.3	mean	3.4	SD	intake: vit A, B1, B2, B6, B12, niacin, folate, C	foods & supplements	4day food record
		adults (non-supplement users only)	vegans	17	41	mean	3.5	SD		foods only	4day food record
Lloyd-Wright 2003	UK	adults	vegans	69	not reported	not reported	not reported	not reported	status: DHA	users & non-users of supplements	n.a.
Lütjohann 2018	Germany	adults	meat-eaters	19	24.9	mean	2	SD	intake: energy, protein	foods only	7d food diary
			vegetarians	35	24.5	mean	2.5	SD			
			vegans	19	27.0	mean	5.5	SD			
Madry 2012	Poland	adults	vegans	20	22.95	mean	0.2	SD	status: vit B12	non-users of supplements	n.a.
			vegans	10	23.00	mean	0.2	SD			
			vegans	10	22.9	mean	0.2	SD			
Majchrzak 2006	Austria	adults	vegetarians	36	34.2	mean	13.6	SD	intake: B1, B2, B6, B12, folate	foods only	24h recall
			vegans	42	30.7	mean	9.9	SD	status: vit B1, B2, B6, folate, B12	users & non-users of supplements	
			meat-eaters	40	38.48	mean	14.8	SD			
Manjari 2001	India	adults	vegetarians	30	31.2	mean	1.1	SD	status: ALA, EPA, DHA	users & non-users of supplements	n.a.
			meat-eaters	44	31.5	mean	1.7	SD			
Mann 2006	Australia	men	vegetarians	43	34.9	mean	9	SD	intake: energy, protein, PUFA, total n-3 FA, ALA, EPA, DHA	foods only	FFQ
			vegans	18	33	mean	7.7	SD	status: PUFA, total n-3 FA, ALA, EPA, DHA	non-users of supplements	
			meat-eaters	78	37.4	mean	8.4	SD			
Mearns 2017	New Zealand	women (South Asian)	vegetarians	33	not reported	not reported	18-50	range	intake: vit B12	foods only	FFQ
			meat-eaters	27	not reported	not reported	18-50	range			
Montalcini 2015	Italy	adults	meat-eaters	26	30.5	mean	6.7	SD	intake: energy, protein	foods only	7d food record
			vegetarians	26	32.6	mean	8.4	SD			
Morad 2005	Israel	adults	vegetarians	17	not reported	not reported	19-60+	range	status: vit B12	users & non-users of supplements	n.a.
Naik 2013	India	young adults	vegetarians	51	27.6	median	26, 30	25th, 75th percentiles	intake: energy, protein, vit B12, folate<br>status: folate, vit B12, iron	foods only	24h recall
Naik 2018	India	adults	vegetarians	119	28.2 (f) 29 (m)	median	27, 32.5 (f)<br>25.5, 33 (m)	25th, 75th percentiles	intake: energy, protein, vit B12, folate	foods & supplements	24 h recall
									status: folate, vit B12	users & non-users of supplements	
Nakamoto 2008	Japan	men	vegetarians	20	45.2	mean	8.3	SD	intake: energy, protein, fiber, vit A, B1, B2, B6, B12, niacin, folate, E, iron, zinc, calcium, magnesium, phosphorus	foods only	3d food record
			meat-eaters	32	44.2	mean	5.2	SD			

Sensitivity: Confidential

Nakamoto 2008a	Japan	women	vegetarians	55	45.9	mean	8.8	SD	intake: energy, protein, fiber, vit A, B1, B2, B6, B12, niacin, folate, E, iron, zinc, calcium, magnesium, phosphorus	foods only	3d food record
Nebl 2019 a&b	Germany	adults	meat-eaters	27	27.4	mean	4.03	SD	intake: energy, protein, fiber, vit A, B1, B2, B6, B12, niacin, folate, E, iron, zinc, calcium, magnesium, phosphorus	foods only	24h recall
			vegetarians	26	27.6	mean	4.31	SD		non-users of supplements	
			vegans	28	27.5	mean	4.24	SD	status: folate, vit B12, D, iron, zinc, calcium, magnesium		
Obeid 2002	Germany & Netherlands	adults	vegetarians	64	not reported	not reported	not reported	not reported	status: folate, vit B12, iron	users & non-users of supplements	n.a.
			vegans	29	not reported	not reported	not reported	not reported			
			semi-vegetarians	20	not reported	not reported	not reported	not reported			
Obeid 2017	Germany	adults	vegetarians	67	49.4	mean	13.3	SD	status: vit B12	users & non-users of supplements	n.a.
			vegans	38	43.5	mean	17.3	SD			
Outila 2000 a&b	Finland	women	vegetarians	6	33	mean	9	SD	intake: calcium	foods only	FFQ (three times)
			vegans	6	33	mean	7	SD	status: vit D, bone markers	non-users of supplements	
			meat-eaters	16	33	mean	7	SD			
Pinto 2017	UK	adults	vegans	23	49	mean	7.9	SD	intake: energy, protein, PUFA, ALA, EPA	foods only	FFQ
			meat-eaters	24	54	mean	9.1	SD	status: vit B12, D	users & non-users of supplements (users of EPA/DHA supplements were excluded)	
Raghunath 2006	India	adults	vegetarians	170	not reported	not reported	20-40	range	intake: iron, zinc, calcium, magnesium	foods only	24 h collection of duplicate food portion
			meat-eaters	80	not reported	not reported	20-40	range			
Rizzo 2013	USA	adults	meat-eaters	33634	59	mean	30-112	range	intake: energy, protein, fiber, PUFA, total n-3 FA, DHA	foods & supplements	FFQ
			semi-vegetarians	4042	59	mean	30-112	range			
			pesco-vegetarians	6583	59	mean	30-112	range			
			vegetarians	21799	59	mean	30-112	range			
			vegans	5694	59	mean	30-112	range			
Rosell 2005	UK	men	vegetarians	231	46.2	mean	12.1	SD	intake: energy, protein, fiber, PUFA	foods only	FFQ
			vegans	232	42.8	mean	13.1	SD	status: ALA, EPA, DHA	non-users of supplements	
			meat-eaters	196	52.2	mean	10.8	SD			
Sanders 2009	UK	men	vegans	57	not reported	not reported	not reported	not reported	intake: ALA, EPA, DHA	foods only	7-d weight record
			meat-eaters	138	not reported	not reported	not reported	not reported			
Sanders 2014	UK	men	vegans	159	not reported	not reported	20-70	range	status: ALA, EPA, DHA	users & non-users of supplements	n.a.
			meat-eaters	164	not reported	not reported	20-70	range			
Salvador 2019	Spain	adults	vegetarians	49	30	median	12	IQR	status: PUFA, total n-3 FA, ALA, EPA, DHA	users & non-users of supplements	FFQ
			vegans	55	31	median	12	IQR			
Sarter 2015	USA	adults	vegans	166	not reported	not reported	20-90	range	status: ALA, EPA, DHA	non-users of supplements	3d food record
Schwarz 2014	Germany	adults	vegans	10	not reported	not reported	not reported	not reported	status: vit B12	non-users of supplements	n.a.
Schlesier 2012	Germany	adults	vegetarians	14	24.5	mean	19-33	range	status: iron	non-users of supplements	n.a.
			meat-eaters	20	22.5	mean	20-29	range			
Schüpbach 2017	Switzerland	adults	vegetarians	53	30.8	mean	7.6	SD	intake: energy, protein, fiber, PUFA, vit A, B1, B2, B6, B12, niacin, folate, E, iron, zinc, calcium, magnesium, phosphorus	foods only	3d weighted food record
			vegans	53	29.9	mean	7.3	SD		non-users of supplements	
			meat-eaters	100	31.6	mean	9.4	SD	status: vit A, B1, B2, B6, niacin, folate, B12, C, E, iron, zinc, iodine, magnesium		
Sellmeyer 2001	USA	older women	low animal protein	207	74.3	mean	5.4	SD	intake: energy, protein, calcium	foods & supplements	FFQ
			moderate animal protein	207	73.2	mean	4.9	SD			
			high animal protein	207	72.5	mean	4.5	SD			

Sensitivity: Confidential

Shridhar 2014	India	adults	vegetarians	2148	41.2	mean	10.2	SD	intake: energy, protein, fiber, vit B12, folate, C, iron, zinc, calcium	foods only	FFQ
			meat-eaters	4407	40.8	mean	10.4	SD			
Śliwińska 2018	Poland	adults	vegetarians	32	29.8	mean	5.9	SD	status: iron	users & non-users of supplements	24 h recall
			vegans	23	32.7	mean	8	SD			
			meat-eaters	36	28.1	mean	11.5	SD			
Sobiecki 2016	UK	adults	meat-eaters	17244	63.3	mean	11.7	SD	intake: energy, protein, fiber, PUFA, vit A, B1, B2, B6, B12, niacin, folate, C, D, E, iron, zinc, calcium, magnesium	foods only	FFQ
			pesco-vegetarians	4531	58.3	mean	11.2	SD			
			vegetarians	6673	56.1	mean	11	SD			
			vegans	803	54.2	mean	11.1	SD			
Somannavar 2012	India	adults	vegetarians	50	43.9	mean	7.2	SD	status: vit A, E	users & non-users of supplements	n.a.
			meat-eaters	50	46.38	mean	6.34	SD			
Su 2006	Taiwan	postmenopausal women	vegetarians	57	59.2	mean	6.4	SD	status: vit B6, folate, B12	users & non-users of supplements	n.a.
			meat-eaters	61	57.7	mean	5.1	SD			
Su 2011	Taiwan	postmenopausal women	vegetarians	49	58.6	mean	6	SD	status: vit B6, folate, B12	users & non-users of supplements (users of vit B12 supplements were excluded)	n.a.
			meat-eaters	41	57.2	mean	5.4	SD			
Vinagre 2013	Brazil	adults	vegans	21	35	mean	10	SD	intake: energy, fiber, PUFA	foods only	3d food record
			vegetarians	29	37	mean	9	SD			
Waldmann 2003	Germany	adults	vegans	98	42.4	median	23.2, 74.3	5th, 95th percentiles	intake: energy, protein, fiber, PUFA, ALA, vit A, B1, B2, B6, B12, niacin, folate, C, E, iron, zinc, calcium, iodine, magnesium, phosphorus	foods only	9-day FFQ (twice)
			vegetarians	56	44.9	median	25.8, 70.7	5th, 95th percentiles			
Waldmann 2004	Germany	adults	vegetarians	45	44.6	mean	15	SD	status: vit B6, folate, B12, iron	users & non-users of supplements (users of vit B12 supplements were excluded)	9d FFQ (twice)
				86	43.8	mean	15.6	SD			9d FFQ (twice)
Waldmann 2005	Germany	adults	vegans	98	43.4	mean	15.4	SD	intake: PUFA, total n-3 FA	foods only	FFQ (twice)
				56	45.7	mean	14.2	SD	status: folate, B12	non-users of supplements	
Welch 2010	UK	adults (all)	vegetarians	250	57.1	mean	6.8	SD	intake: total n-3 FA, EPA, DHA	foods & supplements	7d food record
			vegans	28	53.7	mean	5.6	SD			
			meat-eaters	14144	61.9	mean	7.7	SD			
		adults (non-supplement users only)	vegetarians	25	61.4	mean	9.9	SD	intake: total n-3 FA, EPA, DHA	foods only	7d food record
			vegans	5	54.4	mean	11.8	SD	status: ALA, EPA, DHA	non-users of supplements	
			meat-eaters	2616	64.5	mean	7.96	SD			
Wójciak 2004	Poland	young women	vegetarians	25	not reported	not reported	18-24	range	status: iron, zinc, calcium, magnesium	users & non-users of supplements	24h recall
			meat-eaters	28	not reported	not reported	18-22	range			
Wongprachum 2012	Thailand	adults & children	vegans	234	not reported	not reported	6-45	range	status: iron	users & non-users of supplements	n.a.
Wu 2006	Taiwan	postmenopausal women	vegetarians	25	52.5	mean	4.8	SD	status: vit E, EPA, DHA	users & non-users of supplements	n.a.
Xie 2019	China	adults	vegans	62	33.8	mean	7.1	SD	intake: energy, protein, vit B12, C, calcium, magnesium, phosphorus	foods only	24h recall
			vegetarians	184	32.3	mean	6.3	SD	status: vit D, BMD	non-users of supplements	
			meat-eaters	246	32.1	mean	6.5	SD			
Yen 2008 & 2010	Taiwan	adults	vegetarians	21	34.8	mean	4.5	SD	intake: energy, protein, B1, B2, B12, folate, C, E, iron, zinc, calcium	foods & supplements	3d food record
			meat-eaters	28	35.9	mean	4.1	SD	status: folate, vit B12, iron, BMD		
Yep 2002	Australia	adults	vegetarians	10	not reported	not reported	21–64	range	status: PUFA, total n-3 FA, ALA, EPA, DHA	users & non-users of supplements	n.a.
			meat-eaters	6	not reported	not reported	21–64	range			

References

Al-Delaimy, W.K.; van Kappel, A.L.; Ferrari, P.; Slimani, N.; Steghens, J.-P.; Bingham, S.; Johansson, I.; Wallström, P.; Overvad, K.; Tjønneland, A., et al. Plasma levels of six carotenoids in nine European countries: report from the European Prospective Investigation into Cancer and Nutrition (EPIC). *Public health nutrition* **2004**, *7* , 713-722, doi:10.1079/phn2004598.

Sensitivity: Confidential

Allen, L.H. Causes of vitamin B12 and folate deficiency. *Food and Nutrition Bulletin* **2008**, *29* , S20-33.

Allès, B.; Baudry, J.; Méjean, C.; Touvier, M.; Péneau, S.; Hercberg, S.; Kesse-Guyot, E. Comparison of Sociodemographic and Nutritional Characteristics between Self-Reported Vegetarians, Vegans, and Meat-Eaters from the NutriNet-Santé Study. *Nutrients* **2017**, *9* , 1023, doi:10.3390/nu9091023.

Andrich, D.E.; Filion, M.E.; Woods, M.; Dwyer, J.T.; Gorbach, S.L.; Goldin, B.R.; Adlercreutz, H.; Aubertin-Leheudre, M. Relationship between essential amino acids and muscle mass, independent of habitual diets, in pre- and post-menopausal US women. *Int J Food Sci Nutr* **2011**, *62* , 719-724, doi:10.3109/09637486.2011.573772.

Ball, M.J.; Ackland, M.L. Zinc intake and status in Australian vegetarians. *The British journal of nutrition* **2000**, *83* , 27-33, doi:10.1017/s0007114500000052.

Barghouti, F.F.; Younes, N.A.; Halaseh, L.J.; Said, T.T.; Ghraiz, S.M. High frequency of low serum levels of vitamin 12 among patients attending Jordan University Hospital. *East Mediterr Health J* **2009**, *15* , 853-860.

Bedford, J.L.; Barr, S.I. Diets and selected lifestyle practices of self-defined adult vegetarians from a population-based sample suggest they are more 'health conscious'. *Int J Behav Nutr Phys Act* **2005**, *2* , 4, doi:10.1186/1479-5868-2-4.

Beezhold, B.L.; Johnston, C.S.; Daigle, D.R. Vegetarian diets are associated with healthy mood states: a cross-sectional study in seventh day adventist adults. *Nutrition journal* **2010**, *9* , 26-26, doi:10.1186/1475-2891-9-26.

Bhatti, A.S.; Mahida, V.I.; Gupte, S.C. Iron status of Hindu brahmin, Jain and Muslim communities in Surat, Gujarat. *Indian J Hematol Blood Transfus* **2007**, *23* , 82-87, doi:10.1007/s12288-008-0004-0

Bissoli, L.; Di Francesco, V.; Ballarin, A.; Mandragona, R.; Trespidi, R.; Brocco, G.; Caruso, B.; Bosello, O.; Zamboni, M. Effect of vegetarian diet on homocysteine levels. *Annals of nutrition & metabolism* **2002**, *46* , 73-79, doi:10.1159/000057644.

Boancă, M.M.; Colosi, H.A.; Crăciun, E.C. The impact of the lacto-ovo vegetarian diet on the erythrocyte superoxide dismutase activity: a study in the Romanian population. *European journal of clinical nutrition* **2014**, *68* , 184-188, doi:10.1038/ejcn.2013.179.

Brantsæter, A.L.; Knutsen, H.K.; Johansen, N.C.; Nyheim, K.A.; Erlund, I.; Meltzer, H.M.; Henjum, S. Inadequate Iodine Intake in Population Groups Defined by Age, Life Stage and Vegetarian Dietary Practice in a Norwegian Convenience Sample. *Nutrients* **2018**, *10* , 230, doi:10.3390/nu10020230.

Burns-Whitmore, B.L.; Haddad, E.H.; Sabaté, J.; Jaceldo-Siegl, K.; Tanzman, J.; Rajaram, S. Effect of n-3 fatty acid enriched eggs and organic eggs on serum lutein in free-living lacto-ovo vegetarians. *European journal of clinical nutrition* **2010**, *64* , 1332-1337, doi:10.1038/ejcn.2010.140.

Burns-Whitmore, B.; Haddad, E.; Sabaté, J.; Rajaram, S. Effects of supplementing n-3 fatty acid enriched eggs and walnuts on cardiovascular disease risk markers in healthy free-living lacto-ovo-vegetarians: a randomized, crossover, free-living intervention study. *Nutrition journal* **2014**, *13* , 29-29, doi:10.1186/1475-2891-13-29.

Cade, J.E.; Burley, V.J.; Greenwood, D.C.; Group, U.K.W.s.C.S.S. The UK Women's Cohort Study: comparison of vegetarians, fish-eaters and meat-eaters. *Public health nutrition* **2004**, *7* , 871-878, doi:10.1079/phn2004620.

Carmel, R.; Mallidi, P.V.; Vinarskiy, S.; Brar, S.; Frouhar, Z. Hyperhomocysteinemia and cobalamin deficiency in young Asian Indians in the United States. *Am J Hematol* **2002**, *70* , 107-114, doi:10.1002/ajh.10093.

Chan, J.; Jaceldo-Siegl, K.; Fraser, G.E. Serum 25-hydroxyvitamin D status of vegetarians, partial vegetarians, and nonvegetarians: the Adventist Health Study-2. *The American journal of clinical nutrition* **2009**, *89* , 1686S-1692S, doi:10.3945/ajcn.2009.26736X.

Chang, C.-M.; Chiu, T.H.T.; Chang, C.-C.; Lin, M.-N.; Lin, C.-L. Plant-Based Diet, Cholesterol, and Risk of Gallstone Disease: A Prospective Study. *Nutrients* **2019**, *11* , 335, doi:10.3390/nu11020335.

Chiu, T.H.T.; Huang, H.-Y.; Chiu, Y.-F.; Pan, W.-H.; Kao, H.-Y.; Chiu, J.P.C.; Lin, M.-N.; Lin, C.-L. Taiwanese vegetarians and omnivores: dietary composition, prevalence of diabetes and IFG. *PloS one* **2014**, *9* , e88547-e88547, doi:10.1371/journal.pone.0088547.

Clarys, P.; Deliens, T.; Huybrechts, I.; Deriemaeker, P.; Vanaelst, B.; De Keyzer, W.; Hebbelinck, M.; Mullie, P. Comparison of nutritional quality of the vegan, vegetarian, semi-vegetarian, pesco-vegetarian and omnivorous diet. *Nutrients* **2014**, *6* , 1318-1332, doi:10.3390/nu6031318.

Clarys, P.; Deriemaeker, P.; Huybrechts, I.; Hebbelinck, M.; Mullie, P. Dietary pattern analysis: a comparison between matched vegetarian and omnivorous subjects. *Nutrition journal* **2013**, *12* , 82-82, doi:10.1186/1475-2891-12-82.

Crowe, F.L.; Steur, M.; Allen, N.E.; Appleby, P.N.; Travis, R.C.; Key, T.J. Plasma concentrations of 25-hydroxyvitamin D in meat eaters, fish eaters, vegetarians and vegans: results from the EPIC-Oxford study. *Public Health Nutr* **2011**, *14* , 340-346, doi:10.1017/S1368980010002454.

Damayanti, D.; Jaceldo-Siegl, K.; Beeson, W.L.; Fraser, G.; Oda, K.; Haddad, E.H. Foods and Supplements Associated with Vitamin B(12) Biomarkers among Vegetarian and Non-Vegetarian Participants of the Adventist Health Study-2 (AHS-2) Calibration Study. *Nutrients* **2018**, *10* , 722, doi:10.3390/nu10060722.

Davey, G.K.; Spencer, E.A.; Appleby, P.N.; Allen, N.E.; Knox, K.H.; Key, T.J. EPIC-Oxford: lifestyle characteristics and nutrient intakes in a cohort of 33 883 meat-eaters and 31 546 non meat-eaters in the UK. *Public Health Nutr* **2003**, *6* , 259-269, doi:10.1079/PHN2002430

de Bortoli, M.C.; Cozzolino, S.M.F. Zinc and selenium nutritional status in vegetarians. *Biological trace element research* **2009**, *127* , 228-233, doi:10.1007/s12011-008-8245-1.

Deriemaeker, P.; Aerenhouts, D.; Hebbelinck, M.; Clarys, P. Nutrient based estimation of acid-base balance in vegetarians and non-vegetarians. *Plant Foods Hum Nutr* **2010**, *65* , 77-82, doi:10.1007/s11130-009-0149-5.

Deriemaeker, P.; Alewaeters, K.; Hebbelinck, M.; Lefevre, J.; Philippaerts, R.; Clarys, P. Nutritional status of Flemish vegetarians compared with non-vegetarians: a matched samples study. *Nutrients* **2010**, *2* , 770-780, doi:10.3390/nu2070770.

Deriemaeker, P.; Aerenhouts, D.; De Ridder, D.; Hebbelinck, M.; Clarys, P. Health aspects, nutrition and physical characteristics in matched samples of institutionalized vegetarian and non-vegetarian elderly (> 65yrs). *Nutr Metab (Lond)* **2011**, *8* , 37, doi:10.1186/1743-7075-8-37.

Dyett, P.A.; Sabaté, J.; Haddad, E.; Rajaram, S.; Shavlik, D. Vegan lifestyle behaviors: an exploration of congruence with health-related beliefs and assessed health indices. *Appetite* **2013**, *67* , 119-124, doi:10.1016/j.appet.2013.03.015.

Dyett, P.; Rajaram, S.; Haddad, E.H.; Sabate, J. Evaluation of a validated food frequency questionnaire for self-defined vegans in the United States. *Nutrients* **2014**, *6* , 2523-2539, doi:10.3390/nu6072523.

Elorinne, A.-L.; Alfthan, G.; Erlund, I.; Kivimäki, H.; Paju, A.; Salminen, I.; Turpeinen, U.; Voutilainen, S.; Laakso, J. Food and Nutrient Intake and Nutritional Status of Finnish Vegans and Non-Vegetarians. *PloS one* **2016**, *11* , e0148235-e0148235, doi:10.1371/journal.pone.0148235.

Farmer, B.; Larson, B.T.; Fulgoni, V.L., 3rd; Rainville, A.J.; Liepa, G.U. A vegetarian dietary pattern as a nutrient-dense approach to weight management: an analysis of the national health and nutrition examination survey 1999-2004. *Journal of the American Dietetic Association* **2011**, *111* , 819-827, doi:10.1016/j.jada.2011.03.012.

Fayet, F.; Flood, V.; Petocz, P.; Samman, S. Avoidance of meat and poultry decreases intakes of omega-3 fatty acids, vitamin B12 , selenium and zinc in young women. *Journal of human nutrition and dietetics : the official journal of the British Dietetic Association* **2014**, *27 Suppl 2* , 135-142, doi:10.1111/jhn.12092.

Fokkema, M.R.; Brouwer, D.A.; Hasperhoven, M.B.; Hettema, Y.; Bemelmans, W.J.; Muskiet, F.A. Polyunsaturated fatty acid status of Dutch vegans and omnivores. *Prostaglandins Leukot Essent Fatty Acids* **2000**, *63* , 279-285, doi:10.1054/plef.2000.0215.

Fokkema, M.R.; Brouwer, D.A.J.; Hasperhoven, M.B.; Martini, I.A.; Muskiet, F.A.J. Short-term supplementation of low-dose γ -linolenic acid (GLA), α -linolenic acid (ALA), or GLA plus ALA does not augment LCP ω 3 status of Dutch vegans to an appreciable extent. *Prostaglandins, Leukotrienes and Essential Fatty Acids (PLEFA)* **2000**, *63* , 287-292, doi:10.1054/plef.2000.0216.

Fokkema, M.R.; van Rieke, H.M.; Bauermann, O.J.; Smit, E.N.; Muskiet, F.A. Short-term carnitine supplementation does not augment LCPomega3 status of vegans and lacto-ovo-vegetarians. *J Am Coll Nutr* **2005**, *24* , 58-64, doi:10.1080/07315724.2005.10719444.

Gallego-Narbón, A.; Zapatera, B.; Álvarez, I.; Vaquero, M.P. Methylmalonic Acid Levels and their Relation with Cobalamin Supplementation in Spanish Vegetarians. *Plant Foods Hum Nutr* **2018**, *73* , 166-171, doi:10.1007/s11130-018-0677-y.

Gallego-Narbón, A.; Zapatera, B.; Barrios, L.; Vaquero, M.P. Vitamin B(12) and folate status in Spanish lacto-ovo vegetarians and vegans. *J Nutr Sci* **2019**, *8* , e7-e7, doi:10.1017/jns.2019.2.

Gallego-Narbón, A.; Zapatera, B.; Vaquero, M.P. Physiological and Dietary Determinants of Iron Status in Spanish Vegetarians. *Nutrients* **2019**, *11* , 1734, doi:10.3390/nu11081734.

Gammon, C.S.; von Hurst, P.R.; Coad, J.; Kruger, R.; Stonehouse, W. Vegetarianism, vitamin B12 status, and insulin resistance in a group of predominantly overweight/obese South Asian women. *Nutrition (Burbank, Los Angeles County, Calif.)* **2012**, *28* , 20-24, doi:10.1016/j.nut.2011.05.006.

Geisel, J.; Schorr, H.; Bodis, M.; Isber, S.; Hübner, U.; Knapp, J.-P.; Obeid, R.; Herrmann, W. The vegetarian lifestyle and DNA methylation. *Clin Chem Lab Med* **2005**, *43* , 1164-1169, doi:10.1515/CCLM.2005.202.

Geppert, J.; Kraft, V.; Demmelmair, H.; Koletzko, B. Docosahexaenoic acid supplementation in vegetarians effectively increases omega-3 index: a randomized trial. *Lipids* **2005**, *40* , 807-814, doi:10.1007/s11745-005-1442-9

Geppert, J.; Kraft, V.; Demmelmair, H.; Koletzko, B. Microalgal docosahexaenoic acid decreases plasma triacylglycerol in normolipidaemic vegetarians: a randomised trial. *The British journal of nutrition* **2006**, *95* , 779-786, doi:10.1079/bjn20051720.

Gilsing, A.M.J.; Crowe, F.L.; Lloyd-Wright, Z.; Sanders, T.A.B.; Appleby, P.N.; Allen, N.E.; Key, T.J. Serum concentrations of vitamin B12 and folate in British male omnivores, vegetarians and vegans: results from a cross-sectional analysis of the EPIC-Oxford cohort study. *European journal of clinical nutrition* **2010**, *64* , 933-939, doi:10.1038/ejcn.2010.142.

Goff, L.M.; Bell, J.D.; So, P.W.; Dornhorst, A.; Frost, G.S. Veganism and its relationship with insulin resistance and intramyocellular lipid. *Eur J Clin Nutr* **2005**, *59* , 291-298, doi:10.1038/sj.ejcn.1602076.

Gojda, J.; Patkova, J.; Jacek, M.; Potockova, J.; Trnka, J.; Kraml, P.; Andel, M. Higher insulin sensitivity in vegans is not associated with higher mitochondrial density. *Eur J Clin Nutr* **2013**, *67* , 1310-1315, doi:10.1038/ejcn.2013.202.

Gómez-Donoso, C.; Martínez-González, M.Á.; Martínez, J.A.; Gea, A.; Sanz-Serrano, J.; Perez-Cueto, F.J.A.; Bes-Rastrollo, M. A Provegetarian Food Pattern Emphasizing Preference for Healthy Plant-Derived Foods Reduces the Risk of Overweight/Obesity in the SUN Cohort. *Nutrients* **2019**, *11* , 1553, doi:10.3390/nu11071553.

Gupta, A.K.; Damji, A.; Uppaluri, A. Vitamin B12 deficiency. Prevalence among South Asians at a Toronto clinic. *Can Fam Physician* **2004**, *50* , 743-747.

Haddad, E.H.; Tanzman, J.S. What do vegetarians in the United States eat? *The American journal of clinical nutrition* **2003**, *78* , 626S-632S, doi:10.1093/ajcn/78.3.626S.

Hansen, T.H.; Madsen, M.T.B.; Jørgensen, N.R.; Cohen, A.S.; Hansen, T.; Vestergaard, H.; Pedersen, O.; Allin, K.H. Bone turnover, calcium homeostasis, and vitamin D status in Danish vegans. *European journal of clinical nutrition* **2018**, *72* , 1046-1054, doi:10.1038/s41430-017-0081-y.

Haroon, N.N.; Marwaha, R.K.; Godbole, M.M.; Gupta, S.K. Role of B_{12} and homocysteine status in determining BMD and bone turnover in young Indians. *J Clin Densitom* **2012**, *15* , 366-373, doi:10.1016/j.jocd.2012.01.006.

Harvey, L.J.; Armah, C.N.; Dainty, J.R.; Foxall, R.J.; John Lewis, D.; Langford, N.J.; Fairweather-Tait, S.J. Impact of menstrual blood loss and diet on iron deficiency among women in the UK. *The British journal of nutrition* **2005**, *94* , 557-564, doi:10.1079/bjn20051493.

Hawk, S.N.; Englehardt, K.G.; Small, C. Risks of iron deficiency among vegetarian college women. *Health* **2012**, *04* , 113-119, doi:10.4236/health.2012.43018.

He, J.; Shen, X.; Fang, A.; Song, J.; Li, H.; Guo, M.; Li, K. Association between predominantly plant-based diets and iron status in Chinese adults: a cross-sectional analysis. *The British journal of nutrition* **2016**, *116* , 1621-1632, doi:10.1017/S0007114516003639.

Henjum, S.; Brantsæter, A.L.; Kurniasari, A.; Dahl, L.; Aadland, E.K.; Gjengedal, E.L.F.; Birkeland, S.; Aakre, I. Suboptimal Iodine Status and Low Iodine Knowledge in Young Norwegian Women. *Nutrients* **2018**, *10* , 941, doi:10.3390/nu10070941.

Herrmann, W.; Schorr, H.; Purschwitz, K.; Rassoul, F.; Richter, V. Total homocysteine, vitamin B(12), and total antioxidant status in vegetarians. *Clin Chem* **2001**, *47* , 1094-1101.

Herrmann, W.; Obeid, R.; Schorr, H.; Geisel, J. Functional vitamin B12 deficiency and determination of holotranscobalamin in populations at risk. *Clin Chem Lab Med* **2003**, *41* , 1478-1488, doi:10.1515/CCLM.2003.227.

Herrmann, W.; Schorr, H.; Obeid, R.; Geisel, J. Vitamin B-12 status, particularly holotranscobalamin II and methylmalonic acid concentrations, and hyperhomocysteinemia in vegetarians. *The American journal of clinical nutrition* **2003**, *78* , 131-136, doi:10.1093/ajcn/78.1.131.

Herrmann, W.; Obeid, R.; Schorr, H.; Geisel, J. The usefulness of holotranscobalamin in predicting vitamin B12 status in different clinical settings. *Curr Drug Metab* **2005**, *6* , 47-53, doi:10.2174/1389200052997384.

Herrmann, W.; Obeid, R.; Schorr, H.; Hubner, U.; Geisel, J.; Sand-Hill, M.; Ali, N.; Herrmann, M. Enhanced bone metabolism in vegetarians--the role of vitamin B12 deficiency. *Clin Chem Lab Med* **2009**, *47* , 1381-1387, doi:10.1515/CCLM.2009.302.

Ho-Pham, L.T.; Nguyen, P.L.T.; Le, T.T.T.; Doan, T.A.T.; Tran, N.T.; Le, T.A.; Nguyen, T.V. Veganism, bone mineral density, and body composition: a study in Buddhist nuns. *Osteoporosis international : a journal established as result of cooperation between the European Foundation for Osteoporosis and the National Osteoporosis Foundation of the USA* **2009**, *20* , 2087-2093, doi:10.1007/s00198-009-0916-z.

Ho-Pham, L.T.; Vu, B.Q.; Lai, T.Q.; Nguyen, N.D.; Nguyen, T.V. Vegetarianism, bone loss, fracture and vitamin D: a longitudinal study in Asian vegans and non-vegans. *European journal of clinical nutrition* **2012**, *66* , 75-82, doi:10.1038/ejcn.2011.131.

Hua, N.W.; Stoohs, R.A.; Facchini, F.S. Low iron status and enhanced insulin sensitivity in lacto-ovo vegetarians. *Br J Nutr* **2001**, *86* , 515-519, doi:10.1079/bjn2001421.

Huang, Y.-C.; Chang, S.-J.; Chiu, Y.-T.; Chang, H.-H.; Cheng, C.-H. The status of plasma homocysteine and related B-vitamins in healthy young vegetarians and nonvegetarians. *European journal of nutrition* **2003**, *42* , 84-90, doi:10.1007/s00394-003-0387-5.

Huang, C.-J.; Fan, Y.-C.; Liu, J.-F.; Tsai, P.-S. Characteristics and nutrient intake of Taiwanese elderly vegetarians: evidence from a national survey. *The British journal of nutrition* **2011**, *106* , 451-460, doi:10.1017/S0007114511000195.

Huang, T.; Yu, X.; Shou, T.; Wahlqvist, M.L.; Li, D. Associations of plasma phospholipid fatty acids with plasma homocysteine in Chinese vegetarians. *Br J Nutr* **2013**, *109* , 1688-1694, doi:10.1017/S000711451200356X.

Hung, C.-J.; Huang, P.-C.; Lu, S.-C.; Li, Y.-H.; Huang, H.-B.; Lin, B.-F.; Chang, S.-J.; Chou, H.-F. Plasma homocysteine levels in Taiwanese vegetarians are higher than those of omnivores. *The Journal of nutrition* **2002**, *132* , 152-158, doi:10.1093/jn/132.2.152.

Jin, Y.; Kanaya, A.M.; Kandula, N.R.; Rodriguez, L.A.; Talegawkar, S.A. Vegetarian Diets Are Associated with Selected Cardiometabolic Risk Factors among Middle-Older Aged South Asians in the United States. *The Journal of nutrition* **2018**, *148* , 1954-1960, doi:10.1093/jn/nxy217.

Kajanachumpol, S.; Atamasirikul, K.; Viroonudomphol, D. Iron status among Thai Vegans. *J Med Tech Assoc Thailand* **2011**, *39* , 3929-3936.

Karabudak, E.; Kiziltan, G.; Cigerim, N. A comparison of some of the cardiovascular risk factors in vegetarian and omnivorous Turkish females. *Journal of human nutrition and dietetics : the official journal of the British Dietetic Association* **2008**, *21* , 13-22, doi:10.1111/j.1365-277X.2007.00831.x.

Kazimírová, A.; Barancoková, M.; Krajcovicová-Kudlácková, M.; Volkovová, K.; Staruchová, M.; Valachovicová, M.; Pauková, V.; Blazícek, P.; Wsólová, L.; Dusinská, M. The relationship between micronuclei in human lymphocytes and selected micronutrients in vegetarians and non-vegetarians. *Mutat Res* **2006**, *611* , 64-70, doi:10.1016/j.mrgentox.2006.08.001.

Kim, M.-H.; Choi, M.-K.; Sung, C.-J. Bone mineral density of Korean postmenopausal women is similar between vegetarians and nonvegetarians. *Nutrition Research* **2007**, *27* , 612-617, doi:10.1016/j.nutres.2007.07.006.

Kim, M.H.; Bae, Y.J. Postmenopausal vegetarians' low serum ferritin level may reduce the risk for metabolic syndrome. *Biol Trace Elem Res* **2012**, *149* , 34-41, doi:10.1007/s12011-012-9405-x.

Kapoor, A.; Baig, M.; Tunio, S.A.; Memon, A.S.; Karmani, H. Neuropsychiatric and neurological problems among Vitamin B12 deficient young vegetarians. *Neurosciences (Riyadh)* **2017**, *22* , 228-232, doi:10.17712/nsj.2017.3.20160445.

Kniskern, M.A.; Johnston, C.S. Protein dietary reference intakes may be inadequate for vegetarians if low amounts of animal protein are consumed. *Nutrition* **2011**, *27* , 727-730, doi:10.1016/j.nut.2010.08.024.

Knurick, J.R.; Johnston, C.S.; Wherry, S.J.; Aguayo, I. Comparison of correlates of bone mineral density in individuals adhering to lacto-ovo, vegan, or omnivore diets: a cross-sectional investigation. *Nutrients* **2015**, *7* , 3416-3426, doi:10.3390/nu7053416.

Johnston, C.S.; Bliss, C.; Knurick, J.R.; Scholtz, C. Rapid Eating Assessment for Participants [shortened version] scores are associated with Healthy Eating Index-2010 scores and other indices of diet quality in healthy adult omnivores and vegetarians. *Nutrition journal* **2018**, *17* , 89-89, doi:10.1186/s12937-018-0399-x.

Kornsteiner, M.; Singer, I.; Elmadfa, I. Very low n-3 long-chain polyunsaturated fatty acid status in Austrian vegetarians and vegans. *Annals of nutrition & metabolism* **2008**, *52* , 37-47, doi:10.1159/000118629.

Kotova, N.; Frostne, C.; Abramsson-Zetterberg, L.; Tareke, E.; Bergman, R.; Haghdoost, S.; Paulsson, B.; Törnqvist, M.; Segerbäck, D.; Jenssen, D., et al. Differences in micronucleus frequency and acrylamide adduct levels with hemoglobin between vegetarians and non-vegetarians. *European journal of nutrition* **2015**, *54* , 1181-1190, doi:10.1007/s00394-014-0796-7.

Krajcovicova-Kudlackova, M.; Blazicek, P.; Babinska, K.; Kopcova, J.; Klvanova, J.; Bederova, A.; Magalova, T. Traditional and alternative nutrition—levels of homocysteine and lipid parameters in adults. *Scand J Clin Lab Invest* **2000**, *60* , 657–664.

Krajcovicova-Kudlackova, M.; Ursinyova, M.; Blazicek, P.; Spustova, V.; Ginter, E.; Hladikova, V.; Klvanova, J. Free radical disease prevention and nutrition. *Bratisl Lek Listy* **2003**, *104* , 64-68.

Krajčovičová-Kudláčková, M.; Bučková, K.; Klimeš, I.; Šeboková, E. Iodine Deficiency in Vegetarians and Vegans. *Annals of Nutrition and Metabolism* **2003**, *47* , 183-185, doi:10.1159/000070483.

Krajcovicova-Kudlackova, M.; Blazicek, P.; Mislanova, C.; Valachovicova, M.; Paukova, V.; Spustova, V. Nutritional determinants of plasma homocysteine. *Bratisl Lek Listy* **2007**, *108* , 510-515.

Krajcovicova-Kudlackova, M.; Babinska, K.; Blazicek, P.; Valachovicova, M.; Spustova, V.; Mislanova, C.; Paukova, V. Selected biomarkers of age-related diseases in older subjects with different nutrition. *Bratisl Lek Listy* **2011**, *112* , 610-613.

Krajcovicová-Kudlácková, M.; Valachovicová, M.; Blazícek, P. Seasonal folate serum concentrations at different nutrition. *Cent Eur J Public Health* **2013**, *21* , 36-38.

Kristensen, N.B.; Madsen, M.L.; Hansen, T.H.; Allin, K.H.; Hoppe, C.; Fagt, S.; Lausten, M.S.; Gøbel, R.J.; Vestergaard, H.; Hansen, T., et al. Intake of macro- and micronutrients in Danish vegans. *Nutrition journal* **2015**, *14* , 115-115, doi:10.1186/s12937-015-0103-3.

Krivosíková, Z.; Krajcovicová-Kudlácková, M.; Spustová, V.; Stefíková, K.; Valachovicová, M.; Blazícek, P.; Němcová, T. The association between high plasma homocysteine levels and lower bone mineral density in Slovak women: the impact of vegetarian diet. *European journal of nutrition* **2010**, *49* , 147-153, doi:10.1007/s00394-009-0059-1.

Sensitivity: Confidential

Kuchta, A.; Lebiedzinska, A.; Fijalkowski, M.; Galaska, R.; Kreft, E.; Toton, M.; Czaja, K.; Kozlowska, A.; Cwiklinska, A.; Kortas-Stempak, B., et al. Impact of plant-based diet on lipid risk factors for atherosclerosis. *Cardiol J* **2016**, *23* , 141-148, doi:10.5603/CJ.a2016.0002.

Kwok, T.; Cheng, G.; Woo, J.; Lai, W.K.; Pang, C.P. Independent effect of vitamin B12 deficiency on hematological status in older Chinese vegetarian women. *Am J Hematol* **2002**, *70* , 186-190, doi:10.1002/ajh.10134.

Kwok, T.; Cheng, G.; Lai, W.K.; Poon, P.; Woo, J.; Pang, C.P. Use of fasting urinary methylmalonic acid to screen for metabolic vitamin B12 deficiency in older persons. *Nutrition* **2004**, *20* , 764-768, doi:10.1016/j.nut.2004.06.001.

Kwok, T.; Chook, P.; Qiao, M.; Tam, L.; Poon, Y.K.P.; Ahuja, A.T.; Woo, J.; Celermajer, D.S.; Woo, K.S. Vitamin B-12 supplementation improves arterial function in vegetarians with subnormal vitamin B-12 status. *The journal of nutrition, health & aging* **2012**, *16* , 569-573, doi:10.1007/s12603-012-0036-x.

Leblanc, J.C.; Yoon, H.; Kombadjian, A.; Verger, P. Nutritional intakes of vegetarian populations in France. *European journal of clinical nutrition* **2000**, *54* , 443-449, doi:10.1038/sj.ejcn.1600967.

Lee, H.Y.; Woo, J.; Chen, Z.Y.; Leung, S.F.; Peng, X.H. Serum fatty acid, lipid profile and dietary intake of Hong Kong Chinese omnivores and vegetarians. *European journal of clinical nutrition* **2000**, *54* , 768-773, doi:10.1038/sj.ejcn.1601089.

Lee, Y.; Krawinkel, M. The nutritional status of iron, folate, and vitamin B-12 of Buddhist vegetarians. *Asia Pacific journal of clinical nutrition* **2011**, *20* , 42-49.

Leonard, A.J.; Chalmers, K.A.; Collins, C.E.; Patterson, A.J. The effect of nutrition knowledge and dietary iron intake on iron status in young women. *Appetite* **2014**, *81* , 225-231, doi:10.1016/j.appet.2014.06.021

Leung, A.M.; Lamar, A.; He, X.; Braverman, L.E.; Pearce, E.N. Iodine status and thyroid function of Boston-area vegetarians and vegans. *The Journal of clinical endocrinology and metabolism* **2011**, *96* , E1303-E1307, doi:10.1210/jc.2011-0256.

Li, D.; Sinclair, A.J.; Mann, N.J.; Turner, A.; Ball, M.J. Selected micronutrient intake and status in men with differing meat intakes, vegetarians and vegans. *Asia Pac J Clin Nutr* **2000**, *9* , 18-23, doi:10.1046/j.1440-6047.2000.00129.x.

Lightowler, H.J.; Davies, G.J. Non-starch polysaccharide intake in vegans and the relationship with energy distribution and mineral intakes. *J Hum Nutr Dietet* **2000**, *13* , 443-450.

Lightowler, H.J.; Davies, G.J. Micronutrient intakes in a group of UK vegans and the contribution of self-selected dietary supplements. *J R Soc Promot Health* **2000**, *120* , 117-124, doi:10.1177/146642400012000210.

Lloyd-Wright, Z.; Preston, R.; Gray, R.; Key, T.; Sanders, T. Randomized placebo controlled trial of a daily intake of 200mg docosahexaenoic acid in vegans. *Proc. Nutr. Soc.* **2003**, *62* , 42A.

Lütjohann, D.; Meyer, S.; von Bergmann, K.; Stellaard, F. Cholesterol Absorption and Synthesis in Vegetarians and Omnivores. *Mol Nutr Food Res* **2018**, *62* , e1700689-e1700689, doi:10.1002/mnfr.201700689.

Mądry, E.; Lisowska, A.; Grebowiec, P.; Walkowiak, J. The impact of vegan diet on B-12 status in healthy omnivores: five-year prospective study. *Acta Sci Pol Technol Aliment* **2012**, *11* , 209-212.

Majchrzak, D.; Singer, I.; Männer, M.; Rust, P.; Genser, D.; Wagner, K.H.; Elmadfa, I. B-vitamin status and concentrations of homocysteine in Austrian omnivores, vegetarians and vegans. *Annals of nutrition & metabolism* **2006**, *50* , 485-491, doi:10.1159/000095828.

Manjari, V.; Suresh, Y.; Sailaja Devi, M.M.; Das, U.N. Oxidant stress, anti-oxidants and essential fatty acids in South Indian vegetarians and non-vegetarians. *Prostaglandins Leukot Essent Fatty Acids* **2001**, *64* , 53-59, doi:10.1054/plef.2000.0237.

Mann, N.; Pirotta, Y.; O'Connell, S.; Li, D.; Kelly, F.; Sinclair, A. Fatty acid composition of habitual omnivore and vegetarian diets. *Lipids* **2006**, *41* , 637-646, doi:10.1007/s11745-006-5014-9.

Mearns, G.J.; Rush, E.C. Screening for inadequate dietary vitamin B-12 intake in South Asian women using a nutrient-specific, semi-quantitative food frequency questionnaire. *Asia Pacific journal of clinical nutrition* **2017**, *26* , 1119-1124, doi:10.6133/apjcn.112016.06.

Montalcini, T.; De Bonis, D.; Ferro, Y.; Carè, I.; Mazza, E.; Accattato, F.; Greco, M.; Foti, D.; Romeo, S.; Gulletta, E., et al. High Vegetable Fats Intake Is Associated with High Resting Energy Expenditure in Vegetarians. *Nutrients* **2015**, *7* , 5933-5947, doi:10.3390/nu7075259.

Morad, M.; Gringols, M.; Kandel, I.; Merrick, J. Vitamin B12 deficiency in persons with intellectual disability in a vegetarian residential care community. *ScientificWorldJournal* **2005**, *5* , 58-61, doi:10.1100/tsw.2005.11.

Naik, S.; Bhide, V.; Babhulkar, A.; Mahalle, N.; Parab, S.; Thakre, R.; Kulkarni, M. Daily milk intake improves vitamin B-12 status in young vegetarian Indians: an intervention trial. *Nutrition journal* **2013**, *12* , 136-136, doi:10.1186/1475-2891-12-136.

Naik, S.; Mahalle, N.; Bhide, V. Identification of vitamin B12 deficiency in vegetarian Indians. *The British journal of nutrition* **2018**, *119* , 629-635, doi:10.1017/S0007114518000090.

Nakamoto, K.; Watanabe, S.; Kudo, H.; Tanaka, A. Nutritional characteristics of middle-aged Japanese vegetarians. *J Atheroscler Thromb* **2008**, *15* , 122-129, doi:10.5551/jat.e546.

Nebl, J.; Haufe, S.; Eigendorf, J.; Wasserfurth, P.; Tegtbur, U.; Hahn, A. Exercise capacity of vegan, lacto-ovo-vegetarian and omnivorous recreational runners. *Journal of the International Society of Sports Nutrition* **2019**, *16* , 23-23, doi:10.1186/s12970-019-0289-4.

Nebl, J.; Schuchardt, J.P.; Ströhle, A.; Wasserfurth, P.; Haufe, S.; Eigendorf, J.; Tegtbur, U.; Hahn, A. Micronutrient Status of Recreational Runners with Vegetarian or Non-Vegetarian Dietary Patterns. *Nutrients* **2019**, *11* , 1146, doi:10.3390/nu11051146.

Obeid, R.; Geisel, J.; Schorr, H.; Hübner, U.; Herrmann, W. The impact of vegetarianism on some haematological parameters. *Eur J Haematol* **2002**, *69* , 275-279, doi:10.1034/j.1600-0609.2002.02798.x.

Obeid, R.; Awwad, H.M.; Keller, M.; Geisel, J. Trimethylamine-N-oxide and its biological variations in vegetarians. *European journal of nutrition* **2017**, *56* , 2599-2609, doi:10.1007/s00394-016-1295-9.

Outila, T.A.; Karkkainen, M.U.; Seppanen, R.H.; Lamberg-Allardt, C.J. Dietary intake of vitamin D in premenopausal, healthy vegans was insufficient to maintain concentrations of serum 25-hydroxyvitamin D and intact parathyroid hormone within normal ranges during the winter in Finland. *J Am Diet Assoc* **2000**, *100* , 434-441, doi:10.1016/S0002-8223(00)00134-6.

Outila, T.A.; Lamberg-Allardt, C.J. Ergocalciferol supplementation may positively affect lumbar spine bone mineral density of vegans. *J Am Diet Assoc* **2000**, *100* , 629, doi:10.1016/S0002-8223(00)00184-X.

Pinto, A.M.; Sanders, T.A.B.; Kendall, A.C.; Nicolaou, A.; Gray, R.; Al-Khatib, H.; Hall, W.L. A comparison of heart rate variability, n-3 PUFA status and lipid mediator profile in age- and BMI-matched middle-aged vegans and omnivores - CORRIGENDUM. *Br J Nutr* **2017**, *118* , 877, doi:10.1017/S0007114517002628.

Raghunath, R.; Tripathi, R.M.; Suseela, B.; Bhalke, S.; Shukla, V.K.; Puranik, V.D. Dietary intake of metals by Mumbai adult population. *Sci Total Environ* **2006**, *356* , 62-68, doi:10.1016/j.scitotenv.2005.04.035.

Rizzo, N.S.; Jaceldo-Siegl, K.; Sabate, J.; Fraser, G.E. Nutrient profiles of vegetarian and nonvegetarian dietary patterns. *Journal of the Academy of Nutrition and Dietetics* **2013**, *113* , 1610-1619, doi:10.1016/j.jand.2013.06.349.

Rosell, M.S.; Lloyd-Wright, Z.; Appleby, P.N.; Sanders, T.A.B.; Allen, N.E.; Key, T.J. Long-chain n-3 polyunsaturated fatty acids in plasma in British meat-eating, vegetarian, and vegan men. *The American journal of clinical nutrition* **2005**, *82* , 327-334, doi:10.1093/ajcn.82.2.327.

Sanders, T. DHA status of vegetarians. *Prostaglandins, Leukotrienes and Essential Fatty Acids (PLEFA)* **2009**, *81* .

Sanders, T.A.B. Plant compared with marine n-3 fatty acid effects on cardiovascular risk factors and outcomes: what is the verdict? *The American journal of clinical nutrition* **2014**, *100 Suppl 1* , 453S-458S, doi:10.3945/ajcn.113.071555.

Salvador, A.M.; García-Maldonado, E.; Gallego-Narbón, A.; Zapatera, B.; Vaquero, M.P. Fatty Acid Profile and Cardiometabolic Markers in Relation with Diet Type and Omega-3 Supplementation in Spanish Vegetarians. *Nutrients* **2019**, *11* , 1659, doi:10.3390/nu11071659.

Sarter, B.; Kelsey, K.S.; Schwartz, T.A.; Harris, W.S. Blood docosahexaenoic acid and eicosapentaenoic acid in vegans: Associations with age and gender and effects of an algal-derived omega-3 fatty acid supplement. *Clin Nutr* **2015**, *34* , 212-218, doi:10.1016/j.clnu.2014.03.003.

Schwarz, J.; Dschietzig, T.; Schwarz, J.; Dura, A.; Nelle, E.; Watanabe, F.; Wintgens, K.F.; Reich, M.; Armbruster, F.P. The influence of a whole food vegan diet with Nori algae and wild mushrooms on selected blood parameters. *Clin Lab* **2014**, *60* , 2039-2050, doi:10.7754/clin.lab.2014.140527.

Schlesier, K.; Kühn, B.; Kiehntopf, M.; Winnefeld, K.; Roskos, M.; Bitsch, R.; Böhm, V. Comparative evaluation of green and black tea consumption on the iron status of omnivorous and vegetarian people. *Food Research International* **2012**, *46* , 522-527, doi:10.1016/j.foodres.2011.06.019.

Schüpbach, R.; Wegmüller, R.; Berguerand, C.; Bui, M.; Herter-Aeberli, I. Micronutrient status and intake in omnivores, vegetarians and vegans in Switzerland. *European journal of nutrition* **2017**, *56* , 283-293, doi:10.1007/s00394-015-1079-7.

Sellmeyer, D.E.; Stone, K.L.; Sebastian, A.; Cummings, S.R. A high ratio of dietary animal to vegetable protein increases the rate of bone loss and the risk of fracture in postmenopausal women. *Am J Clin Nutr* **2001**, *73* , 118–122.

Shridhar, K.; Dhillon, P.K.; Bowen, L.; Kinra, S.; Bharathi, A.V.; Prabhakaran, D.; Reddy, K.S.; Ebrahim, S. Nutritional profile of Indian vegetarian diets--the Indian Migration Study (IMS). *Nutrition journal* **2014**, *13* , 55-55, doi:10.1186/1475-2891-13-55.

Śliwińska, A.; Luty, J.; Aleksandrowicz-Wrona, E.; Małgorzewicz, S. Iron status and dietary iron intake in vegetarians. *Adv Clin Exp Med* **2018**, *27* , 1383-1389, doi:10.17219/acem/70527.

Sobiecki, J.G.; Appleby, P.N.; Bradbury, K.E.; Key, T.J. High compliance with dietary recommendations in a cohort of meat eaters, fish eaters, vegetarians, and vegans: results from the European Prospective Investigation into Cancer and Nutrition-Oxford study. *Nutrition research (New York, N.Y.)* **2016**, *36* , 464-477, doi:10.1016/j.nutres.2015.12.016.

Somannavar, M.S.; Kodliwadmath, M.V. Correlation between oxidative stress and antioxidant defence in South Indian urban vegetarians and non-vegetarians. *Eur Rev Med Pharmacol Sci* **2012**, *16* , 351-354.

Su, T.-C.; Jeng, J.-S.; Wang, J.-D.; Torng, P.-L.; Chang, S.-J.; Chen, C.-F.; Liau, C.-S. Homocysteine, circulating vascular cell adhesion molecule and carotid atherosclerosis in postmenopausal vegetarian women and omnivores. *Atherosclerosis* **2006**, *184* , 356-362, doi:10.1016/j.atherosclerosis.2005.04.022.

Su, T.C.; Torng, P.L.; Jeng, J.S.; Chen, M.F.; Liau, C.S. Arterial function of carotid and brachial arteries in postmenopausal vegetarians. *Vasc Health Risk Manag* **2011**, *7* , 517-523, doi:10.2147/VHRM.S18881.

Vinagre, J.C.; Vinagre, C.G.; Pozzi, F.S.; Slywitch, E.; Maranhao, R.C. Metabolism of triglyceride-rich lipoproteins and transfer of lipids to high-density lipoproteins (HDL) in vegan and omnivore subjects. *Nutr Metab Cardiovasc Dis* **2013**, *23* , 61-67, doi:10.1016/j.numecd.2011.02.011.

Waldmann, A.; Koschizke, J.W.; Leitzmann, C.; Hahn, A. Dietary intakes and lifestyle factors of a vegan population in Germany: results from the German Vegan Study. *European journal of clinical nutrition* **2003**, *57* , 947-955, doi:10.1038/sj.ejcn.1601629.

Waldmann, A.; Koschizke, J.W.; Leitzmann, C.; Hahn, A. Homocysteine and cobalamin status in German vegans. *Public health nutrition* **2004**, *7* , 467-472, doi:10.1079/PHN2003540.

Waldmann, A.; Koschizke, J.W.; Leitzmann, C.; Hahn, A. German vegan study: diet, life-style factors, and cardiovascular risk profile. *Annals of nutrition & metabolism* **2005**, *49* , 366-372, doi:10.1159/000088888.

Welch, A.A.; Shakya-Shrestha, S.; Lentjes, M.A.; Wareham, N.J.; Khaw, K.T. Dietary intake and status of n-3 polyunsaturated fatty acids in a population of fish-eating and non-fish-eating meat-eaters, vegetarians, and vegans and the product-precursor ratio [corrected] of alpha-linolenic acid to long-chain n-3 polyunsaturated fatty acids: results from the EPIC-Norfolk cohort. *Am J Clin Nutr* **2010**, *92* , 1040-1051, doi:10.3945/ajcn.2010.29457.

Wójciak, R.W.; Krejpcio, Z.; Czlapka-Matyasik, M.; Jeszka, J. Comparison of the hair bioelements in vegeterian and non-vegeterian women. *Trace Elements and Electrolytes* **2004**, *21* , 141-144, doi:10.5414/tep21141.

Wongprachum, K.; Sanchaisuriya, K.; Sanchaisuriya, P.; Siridamrongvattana, S.; Manpeun, S.; Schlep, F.P. Proxy indicators for identifying iron deficiency among anemic vegetarians in an area prevalent for thalassemia and hemoglobinopathies. *Acta Haematol* **2012**, *127* , 250-255, doi:10.1159/000337032.

Wu, W.H.; Lu, S.C.; Wang, T.F.; Jou, H.J.; Wang, T.A. Effects of docosahexaenoic acid supplementation on blood lipids, estrogen metabolism, and in vivo oxidative stress in postmenopausal vegetarian women. *European journal of clinical nutrition* **2006**, *60* , 386-392, doi:10.1038/sj.ejcn.1602328.

Xie, L.; Wang, B.; Cui, X.; Tang, Q.; Cai, W.; Shen, X. Young adult vegetarians in Shanghai have comparable bone health to omnivores despite lower serum 25(OH) vitamin D in vegans: a cross-sectional study. *Asia Pacific journal of clinical nutrition* **2019**, *28* , 383-388, doi:10.6133/apjcn.201906_28(2).0021.

Yen, C.-E.; Yen, C.-H.; Huang, M.-C.; Cheng, C.-H.; Huang, Y.-C. Dietary intake and nutritional status of vegetarian and omnivorous preschool children and their parents in Taiwan. *Nutrition research (New York, N.Y.)* **2008**, *28* , 430-436, doi:10.1016/j.nutres.2008.03.012.

Yen, C.-E.; Yen, C.-H.; Cheng, C.-H.; Huang, Y.-C. Vitamin B-12 status is not associated with plasma homocysteine in parents and their preschool children: lacto-ovo, lacto, and ovo vegetarians and omnivores. *Journal of the American College of Nutrition* **2010**, *29* , 7-13, doi:10.1080/07315724.2010.10719811.

Yep, Y.L.; Li, D.; Mann, N.J.; Bode, O.; Sinclair, A.J. Bread enriched with microencapsulated tuna oil increases plasma docosahexaenoic acid and total omega-3 fatty acids in humans. *Asia Pacific J Clin Nutr* **2002**, *11* , 285–291.

Sensitivity: Confidential
