# Supplementary material for: Nutrient Intake and Status in Adults Consuming Plant-Based Diets Compared to Meat-Eaters: A Systematic Review
Source: Nutrients. 2021 Dec 23;14(1):29. doi: 10.3390/nu14010029 (PMC8746448; doi:10.3390/nu14010029)
Supplement: Supplementary file 1 [file nutrients-14-00029-s001.zip › Supplementary table S2_Status descriptives.pdf]

Supplementary Table S2: Nutritional status, hemoglobin, and bone marker data per dietary pattern

	all studies			Studies among non-users of supplements			Studies among users & non-users of supplements		
	meat eaters	vegetarians	vegans	meat eaters	vegetarians	vegans	meat eaters	vegetarians	vegans
Vitamin B12 (pmol/l)									
number of studies	34	42	21	19	24	8	15	18	13
mean	309.0	218.0	221.9	301.4	205.7	200.3	318.6	234.4	358.0
median	309.6	215.5	203.2	310.9	204.1	195.0	308.8	225.3	203.2
min	113.4	108.5	122.0	113.4	108.5	138.7	229.3	145.0	122.0
max	445.0	354.0	358.0	403.5	326.0	269.3	445.0	354.0	358.0
p25	278.6	177.0	159.0	275.75	169.4	156.1	279.4	210.7	177.3
p75	340.5	246.7	275.7	327.5	246.2	245.4	352.1	259.0	296.9
Folate (nmol/l)									
number of studies	32	36	14	17	19	5	15	17	9
mean	19.0	23.6	29.2	17.1	21.3	23.8	21.2	26.2	32.2
median	18.4	22.6	33.1	18.0	20.1	17.5	22.7	24.7	35.0
min	7.6	9.6	15.6	7.6	9.6	15.6	11.9	17.4	20.2
max	29.2	39.4	40.7	25.1	39.4	36.0	29.2	38.4	40.7
p25	13.8	19.1	20.7	12.8	15.4	16.8	16.5	19.8	22.7
p75	24.4	28.1	21.2	20.0	24.1	33.1	26.5	32.9	37.9
Vitamin B6 (nmol/l)									
number of studies	11	10	4	4	4	0	7	6	4
mean	66.2	69.3	60.1	50.9	45.0	n.a.	76.4	85.5	60.1
median	65.3	59.0	59.0	48.3	46.8	n.a.	67.3	70.1	59.0
min	15.9	24.1	14.0	21.2	28.0	n.a.	15.9	24.1	14.0
max	149.2	167.5	108.5	85.9	58.5	n.a.	149.2	167.5	108.5
p25	36.4	46.6	14.0	29.2	41.8	n.a.	54.0	59.6	46.4
p75	81.6	75.2	108.5	69.9	50.0	n.a.	98.7	111.5	72.7
Vitamin C (µmol/l)									
number of studies	7	7	3	4	4	1	3	3	2
mean	44.9	62.7	61.9	46.7	66.7	71.8	42.4	57.5	57.0
median	45.1	61.0	71.8	50.0	63.6	71.8	44.9	61.0	57.0
min	29.8	33.6	37.1	31.5	58.0	71.8	29.8	33.6	37.1
max	55.4	81.5	76.8	55.4	81.5	71.8	52.6	77.9	76.8
p25	38.2	58.1	54.5	41.7	58.2	n.a.	37.3	47.3	47.0
p75	53.8	73.4	74.3	55.0	72.1	n.a.	48.7	69.5	66.9
Vitamin D (µg/l)									
number of studies	12	9	9	5	4	4	7	5	5
mean	26.2	22.7	21.9	22.4	19.6	20.1	29.0	25.2	23.4
median	26.5	26.1	22.3	22.0	17.6	20.1	30.8	26.4	22.3
min	14.7	13.6	10.8	14.7	13.6	10.8	17.6	16.4	15.0
max	38.4	30.7	34.5	32.9	29.5	29.5	38.4	30.7	34.5
p25	20.7	17.0	17.7	15.6	16.5	16.0	24.0	26.1	22.2
p75	31.9	26.4	22.9	26.5	20.7	24.2	33.9	26.4	22.9
Vitamin E (µmol/l)									
number of studies	6	7	4	3	4	2	3	3	2
mean	25.4	25.5	20.5	25.3	23.2	17.1	25.6	28.6	24.0
median	26.3	24.2	33.8	26.0	21.8	17.1	26.6	30.0	24.0
min	12.5	12.7	12.1	12.5	12.7	12.1	22.2	24.2	16.8
max	37.4	36.6	31.2	37.4	36.6	22.1	27.9	31.6	31.2
p25	23.2	21.8	21.8	19.2	18.9	14.6	24.4	27.1	20.4
p75	27.6	30.8	30.8	31.7	26.2	19.6	27.2	30.8	27.6
ß-caroten (µmol/l)									
number of studies	3	4	1	1	2	0	2	2	1
mean	0.8	0.4	0.8	0.2	0.4	n.a.	1.1	0.4	0.8
median	0.3	0.4	0.8	0.2	0.4	n.a.	1.1	0.4	0.8
min	0.2	0.2	0.8	0.2	0.24	n.a.	0.3	0.3	0.8
max	1.9	0.6	0.8	0.2	0.56	n.a.	1.9	0.4	0.8
p25	0.3	0.3	n.a.	n.a.	0.32	n.a.	0.7	0.3	n.a.
p75	1.1	0.4	n.a.	n.a.	0.48	n.a.	1.5	0.4	n.a.

	all studies			Studies among non-users of supplements			Studies among users & non-users of supplements		
	meat eaters	vegetarians	vegans	meat eaters	vegetarians	vegans	meat eaters	vegetarians	vegans
Ferritin (µg/l)									
number of studies	14	14	5	9	9	1	5	5	4
mean	55.5	33.8	31.3	47.2	28.8	50.0	70.5	42.0	26.6
median	43.3	31.9	32.1	41.6	28.0	50.0	78.8	37.1	30.2
min	12.9	12.6	13.4	12.9	12.6	50.0	21.1	20.5	13.4
max	120.7	73.4	50.0	120.7	48.0	50.0	103.5	73.4	32.7
p25	29.9	24.2	28.3	28.4	23.5	n.a.	62.2	31.2	24.6
p75	77.1	38.3	32.7	44.3	33.1	n.a.	86.7	47.9	32.3
Zinc (mg/l)									
number of studies	6	7	3	3	4	1	3	3	2
mean	0.90	0.81	0.79	0.91	0.80	0.72	0.89	0.90	0.82
median	0.92	0.78	0.80	0.93	0.77	0.72	0.91	0.88	0.82
min	0.9	0.7	0.7	0.85	0.66	0.72	0.85	0.87	0.80
max	1.0	1.0	0.8	0.95	0.99	0.72	0.92	0.96	0.84
p25	0.87	0.76	0.76	0.89	0.74	n.a.	0.88	0.88	0.81
p75	0.93	0.88	0.82	0.94	0.83	n.a.	0.92	0.92	0.83
Iodine (µg/l)									
number of studies	5	5	5	0	0	0	5	5	5
mean	111.2	102.8	105.0	n.a.	n.a.	n.a.	111.2	102.8	105.0
median	90.8	100.0	62.3	n.a.	n.a.	n.a.	90.8	100.0	62.3
min	47.2	39.3	13.2	n.a.	n.a.	n.a.	47.2	39.3	13.2
max	216.0	172.0	282.1	n.a.	n.a.	n.a.	216.0	172.0	282.1
p25	75.6	80.8	38.3	n.a.	n.a.	n.a.	75.6	80.8	38.3
p75	126.5	121.9	129.0	n.a.	n.a.	n.a.	126.5	121.9	129.0
Hemoglobin (g/l)									
number of studies	10	14	8	6	8	3	4	6	5
mean	139.2	135.6	140.2	139.2	135.4	141.8	139.2	135.9	139.2
median	138.8	136.0	140.1	138.5	135.5	143.3	138.8	136.2	139.8
min	130.0	126.7	135.0	130.0	126.7	135.0	136.3	130.0	135.0
max	149.6	146.0	147.0	149.6	146.0	147.0	143.0	140.6	141.0
p25	135.7	130.9	138.5	134.3	129.3	139.2	138.0	134.3	139.7
p75	142.6	138.8	141.6	144.1	139.6	145.2	140.0	138.2	140.4
PTH (pmol/l)									
number of studies	4	4	3	3	3	3	1	1	0
mean	4.04	4.91	5.21	3.95	4.94	5.21	4.30	4.86	n.a.
median	4.36	4.86	5.21	4.43	4.94	5.21	4.30	4.86	n.a.
min	2.86	4.35	4.89	2.86	4.35	4.89	4.30	4.86	n.a.
max	4.55	5.52	5.43	4.55	5.52	5.43	4.30	4.86	n.a.
p25	3.94	4.61	5.09	3.65	4.64	5.09	n.a.	n.a.	n.a.
p75	4.43	5.19	5.32	4.46	5.23	5.32	n.a.	n.a.	n.a.
CTX (ng/ml)									
number of studies	5	4	2	4	3	2	1	1	0
mean	0.46	0.50	0.55	0.46	0.51	0.55	0.48	0.49	n.a.
median	0.48	0.54	0.55	0.46	0.59	0.55	0.48	0.49	n.a.
min	0.30	0.27	0.53	0.30	0.27	0.53	0.48	0.49	n.a.
max	0.61	0.66	0.56	0.61	0.66	0.56	0.48	0.49	n.a.
p25	0.43	0.43	0.53	0.40	0.43	0.54	n.a.	n.a.	n.a.
p75	0.48	0.61	0.56	0.51	0.63	0.55	n.a.	n.a.	n.a.
Osteocalcin (µg/l)									
number of studies	4	4	4	4	4	4	0	0	0
mean	22.5	24.9	27.9	22.5	24.9	27.9	n.a.	n.a.	n.a.
median	23.0	26.2	27.9	23.0	26.2	27.9	n.a.	n.a.	n.a.
min	17.67	19.17	23.67	17.7	19.2	23.7	n.a.	n.a.	n.a.
max	26.10	29.37	32.17	26.1	29.4	32.2	n.a.	n.a.	n.a.
p25	21.7	22.7	25.8	21.7	22.7	25.8	n.a.	n.a.	n.a.
p75	23.8	27.8	30.0	23.8	27.8	30.0	n.a.	n.a.	n.a.
BMD l. spine (g/cm2)									
number of studies	4	4	1	3	3	1	1	1	0
mean	0.97	0.95	1.03	1.03	1.01	1.03	0.79	0.77	n.a.
median	0.95	0.94	1.03	1.10	1.08	1.03	0.79	0.77	n.a.
min	0.79	0.77	1.03	0.81	0.81	1.03	0.79	0.77	n.a.
max	1.18	1.14	1.03	1.18	1.14	1.03	0.79	0.77	n.a.
p25	0.80	0.80	n.a.	0.95	0.94	n.a.	n.a.	n.a.	n.a.
p75	1.12	1.09	n.a.	1.14	1.11	n.a.	n.a.	n.a.	n.a.

n.a. not assessed
